# Virtual Screening and *In Vitro* Experimental Verification of LuxS Inhibitors for *Escherichia coli* O157:H7

**DOI:** 10.1128/spectrum.03502-22

**Published:** 2023-02-21

**Authors:** Yu-Bin Bai, Xiao-Rong Yang, Bing Li, Xu-Zheng Zhou, Wei-Wei Wang, Fu-Sheng Cheng, Ji-Yu Zhang

**Affiliations:** a Key Laboratory of New Animal Drug Project of Gansu Province, Lanzhou, Gansu, People’s Republic of China; b Key Laboratory of Veterinary Pharmaceutical Development, Ministry of Agriculture, Lanzhou, Gansu, People’s Republic of China; c Lanzhou Institute of Husbandry and Pharmaceutical Sciences, Chinese Academy of Agricultural Sciences, Lanzhou, Gansu, People’s Republic of China; Migal-Galilee Research Institute

**Keywords:** LuxS, *Escherichia coli* O157:H7, virtual screening, quorum sensing, inhibitors, biofilm

## Abstract

Enterohemorrhagic Escherichia coli O157:H7 is an important foodborne pathogen that forms biofilms. In this study, three quorum-sensing (QS) inhibitors (M414-3326, 3254-3286, and L413-0180) were obtained through virtual screening, and their *in vitro* antibiofilm activities were validated. Briefly, the three-dimensional structure model of LuxS was constructed and characterized using the SWISS-MODEL. High-affinity inhibitors were screened from the ChemDiv database (1,535,478 compounds) using LuxS as a ligand. Five compounds (L449-1159, L368-0079, M414-3326, 3254-3286, and L413-0180) with a good inhibitory effect (50% inhibitory concentration <10 μM) on type II QS signal molecule autoinducer-2 (AI-2) were obtained using a AI-2 bioluminescence assay. The absorption, distribution, metabolism, excretion, and toxicity (ADMET) properties predicated that the five compounds had high intestinal absorption levels (high) and plasma protein binding (absorbent strong) and did not inhibit the metabolism of CYP2D6 metabolic enzymes. In addition, molecular dynamics simulation showed that compounds L449-1159 and L368-0079 could not stably bind with LuxS. Thus, these compounds were excluded. Furthermore, surface plasmon resonance results showed that the three compounds could specifically bind to LuxS. IN addition, the three compounds could effectively inhibit the biofilm formation without affecting the growth and metabolism of the bacteria. Finally, the reverse transcription-quantitative PCR results showed that the three compounds downregulated the expression of the LuxS gene. Overall, these results revealed that the three compounds obtained through virtual screening could inhibit biofilm formation of E. coli O157:H7 and are potential LuxS inhibitors that can be used to treat E. coli O157:H7 infections.

**IMPORTANCE** E. coli O157:H7 is a foodborne pathogen of public health importance. Quorum sensing (QS) is a form of bacterial communication that can regulate various group behaviors, including biofilm formation. Here, we identified three QS AI-2 inhibitors (M414-3326, 3254-3286, and L413-0180) that can stably and specifically bind to LuxS protein. The three QS AI-2 inhibitors inhibited biofilm formation without affecting the growth and metabolic activity of E. coli O157:H7. The three QS AI-2 inhibitors are promising agents for treating E. coli O157:H7 infections. Further studies to identify the mechanism of the three QS AI-2 inhibitors are needed to develop new drugs to overcome antibiotic resistance.

## INTRODUCTION

Escherichia coli O157: H7 is an enterohemorrhagic bacterial strain and foodborne pathogen causing diarrhea, hemorrhagic colitis, and even hemolytic-uremic syndrome in humans. The disease is transmitted via the fecal-oral route in contaminated water or foods and through feces from one individual to another (1). According to the Centers for Disease Control and Prevention, over 63,000 illnesses are caused by foodborne E. coli O157:H7, leading to over 2,100 hospitalizations and deaths yearly. The bacterium causes economic losses approximated at $405 million annually due to medical expenses, death, and productivity losses (2, 3).

**FIG 1:**
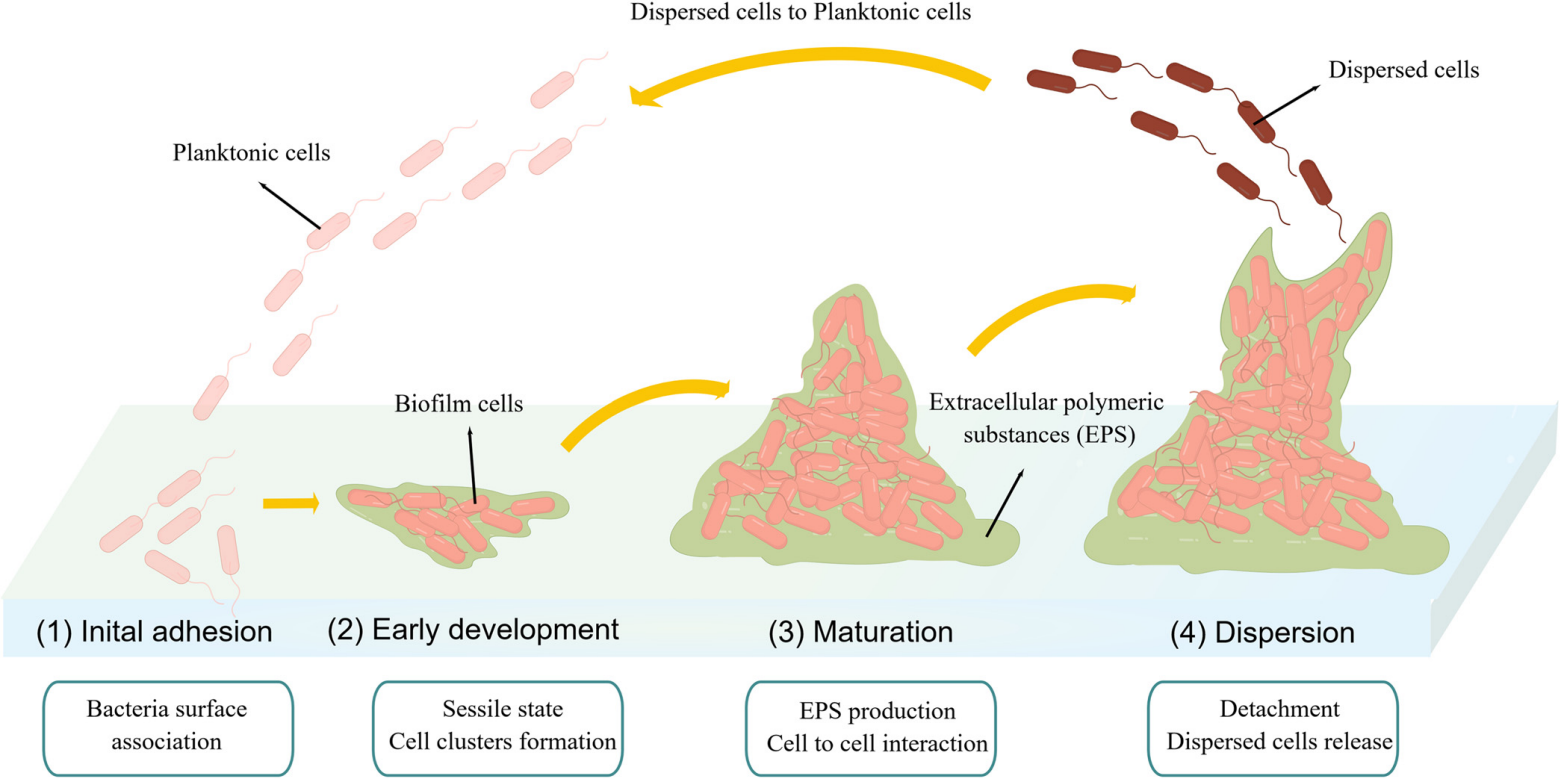
Procedures used for biofilm development.

A biofilm is an aggregate of microorganisms that adhere to a substrate, encapsulated within a self-produced matrix of exopolysaccharides, proteins, and extracellular DNA (4–7). The biofilm formation process mainly includes four stages: (i) initial adhesion (reversible), (ii) early development of biofilm (irreversible), (iii) maturation, and (iv) dispersion (8). During the initial adhesion of biofilm formation, the E. coli attaches to the substratum using flagella (Fig. 1, stage 1). Flagellum synthesis is inhibited during early development. The planktonic cells become sessile and form cell clusters (Fig. 1, stage 2). Once the cells are firmly attached to the surface, they accumulate through intercellular interactions, secrete extracellular matrix, and provide a three-dimensional (3D) structure for the biofilm (Fig. 1, stage 3). When the cells are fully mature, they detach from the mature biofilm, transform into a planktonic state, and spread to form a new biofilm (Fig. 1, stage 4). E. coli O157:H7 can form biofilm on biotic and abiotic surfaces, such as stainless glass, meat, and the colon (9, 10). A pathogenic biofilm can pose serious health risks to humans because of its antimicrobial resistance (11, 12). Antivirulence therapy targeting biofilm is a useful method (13–17), which can reduce pathogenicity without affecting bacterial growth, thus reducing the emergence and spread of resistant mutants (17–20).

**FIG 2:**
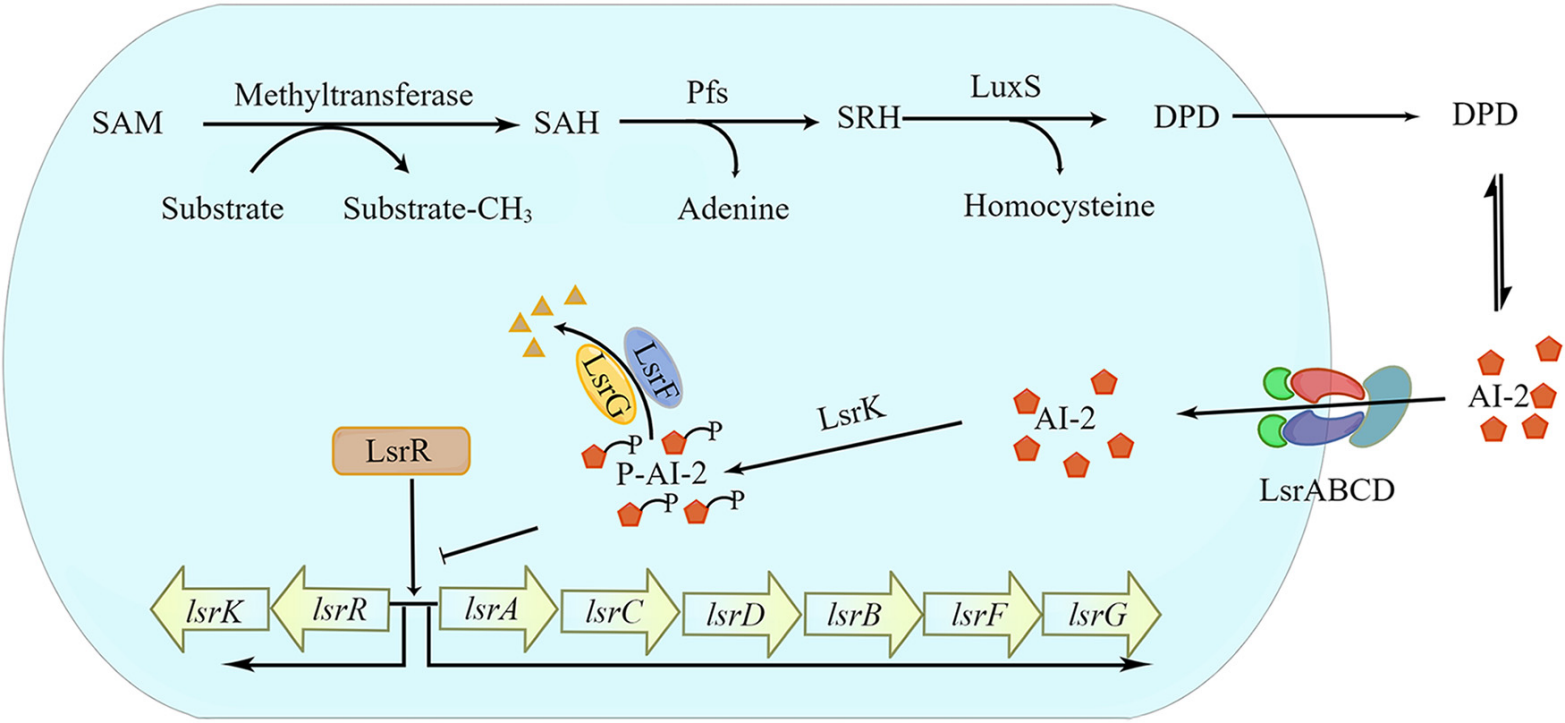
Biosynthesis and transport of AI-2 in E. coli.

Quorum sensing (QS) is a cellular mechanism mediated by autoinducers that allows bacteria to organize their behavior depending on their density (21). QS regulates different pathogenic processes like secretion of virulence factors (22–24), biofilm formation (25–27), and antibiotic sensitivity (28, 29), but it does not result in the development of resistance (30–32). It has been reported that autoinducer-2 (AI-2) is the only QS molecule produced by E. coli. Its production and uptake affect biofilm formation, virulence, and the motility of E. coli (33). *S*-Adenosylhomocysteine (SAH), an intermediate of *S*-adenosylmethionine (SAM), is catalyzed by *S*-adenosylhomocysteine nucleosidase (Pfs) to produce *S*-ribosylhomocysteine (SRH). LuxS converts SRH to 4,5-dihydroxy 2,3-pentanedione (DPD) and homocysteine. DPD is unstable and finally forms AI-2 (Fig. 2) (34). The Lsr system is responsible for the detection, uptake, and signal transduction of AI-2 in E. coli (Fig. 2) (35). During early bacterial growth, extracellular AI-2 concentration is low, and LsrR inhibits the expression of the Lsr system. As the bacteria grow and the extracellular AI-2 accumulates, AI-2 binds to the periplasmic binding protein, LsrB. AI-2 is further internalized by Lsr transporters formed by two transmembrane proteins (LsrC and LsrD) and ATP-binding protein (LsrA) and then phosphorylated by LsrK. P-AI-2 binds to LsrR, leading to the disinhibition of the Lsr operon, LsrR, and LsrK, and the Lsr-related transport starts. Due to this positive-feedback loop, AI-2 input increases, and the extracellular level of AI-2 is rapidly depleted. The LsrG and LsrF degrade P-AI-2. LuxS is a potential target for new drugs because it exists in many bacterial species but not in mammals (35–37). According to the structural classification of proteins, the LuxS enzyme belongs to the LuxS/MPP-like metallohydrolase superfamily. In addition, studies on the LuxS gene have shown that the gene is highly conserved in different species but has no homology with other genes (38). The crystal structure of LuxS protein obtained for the first time (39, 40) indicated that LuxS protein is a homodimer, retaining an eight-stranded β-barrel surrounded by six α-helices. The active site consists of zinc ions coordinated by highly conserved residues His54, His58, and Cys126. Also, the entry of active sites is restricted and triggered by conformational changes in the protein, involving residues 125 to 131 and residues around the N terminus. A notable feature of this protein is that it is one of the few enzymes that can cleave thioether bonds without using redox cofactors (41). Currently, many inhibitors against LuxS have been explored. For example, Meng et al. screened four compounds that bind well with LuxS from 72 natural products through virtual screening, including norathyriol, mangiferin, baicalein, and kaempferol, which can effectively inhibit the production of AI-2 (42). Ali et al. predicted the structure of LuxS from Aeromonas hydrophila, characterized its structure and function, and screened inhibitors (−)-dimethyl 2,3-*O*-isopropylidene-l-tartrate from the ZINC database through high-throughput screening (43). Mina et al. reviewed the synthesized LuxS inhibitors, *S*-ribosylhomocysteine analogs, which can compete with the native signaling process to inhibit the production of signaling molecules (44).

Virtual screening technology has been widely used in drug discovery (42, 45, 46). The present study screened quorum-sensing inhibitors (QSIs) from the ChemDiv database (1,535,478 compounds). The biological processes, including AI-2 production, cytotoxicity, and biofilm formation regulated by QSIs, were identified using an *in vitro* assay. This study explored the potential of E. coli O157:H7 infection treatment by inhibiting LuxS.

## RESULTS AND DISCUSSION

### Homology modeling of LuxS and model evaluation.

**FIG 3:**
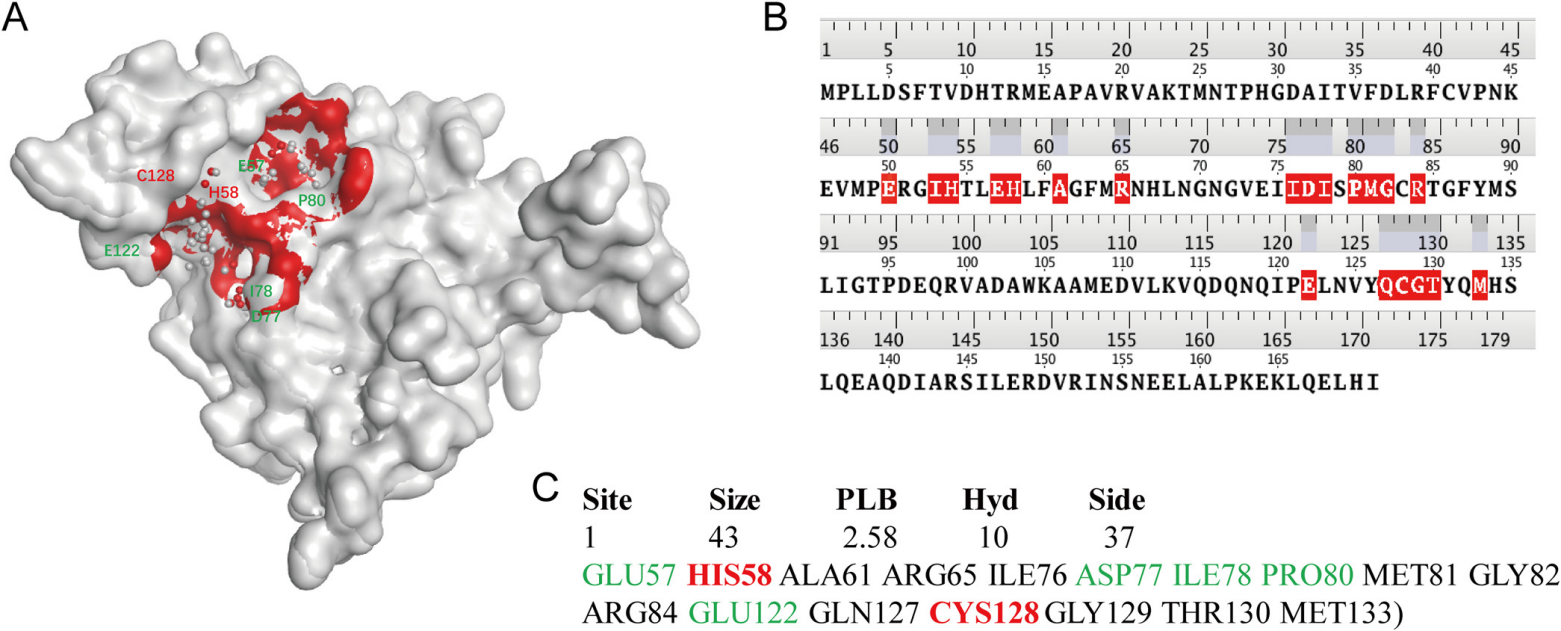
Structure of LuxS protein of E. coli. (A) The pocket (red) of LuxS protein binds to a small-molecule pocket. The length, width, and depth of the pocket are 21.33, 26.50, and 18.67 Å, respectively. (B) The amino acids in the small-molecule binding pocket of the sequence are labeled red. (C) In the pocket attribute, the red part is the metal-binding site, and the green part was the methionine-binding site.

This study used chain B of 5E68 as a template for the homology modeling of LuxS for E. coli. The query coverage was 100%, whereas the sequence homology was 93.57%. The qualitative model energy analysis (QMEAN) value was 0.50, indicating that the 3D structure model of LuxS was standard (see Fig. S1 in the supplemental material). The 3D structure of LuxS for E. coli is shown in Fig. 3A. The root mean square deviation (RMSD) value of 171 amino acids in this model and the template model was 1.53 Å, indicating that they had highly similar 3D structures.

### LuxS pocket of *E. coli*.

We found that methionine binds to LuxS, and the binding sites are E57, H58, D77, I78, S79, P80, and E122 (see Fig. S2). A small-molecule binding pocket (indicated in red in Fig. 3A) in LuxS was identified using the SiteFinder module in MOE (v11.16.0.0), which contained up to 37 atoms and had a score of 2.58. The pocket contained amino acids GLU50, ILE53, HIS54, GLU57, HIS58, ALA61, ARG65, ILE76, ASP77, ILE78, PRO80, MET81, GLY82, ARG84, GLU122, GLN127, CYS128, GLY129, THR130, and MET133; methionine binding sites (E57, H58, D77, I78, P80, and E122); and metal binding sites (H54, H58, and C128) (Fig. 3B and C). Therefore, we speculated that the compounds that bind to the pocket potentially interfered with LuxS binding substrates or metal ions and that these high-affinity compounds were potentially LuxS inhibitors.

### Molecular docking score.

**FIG 4:**
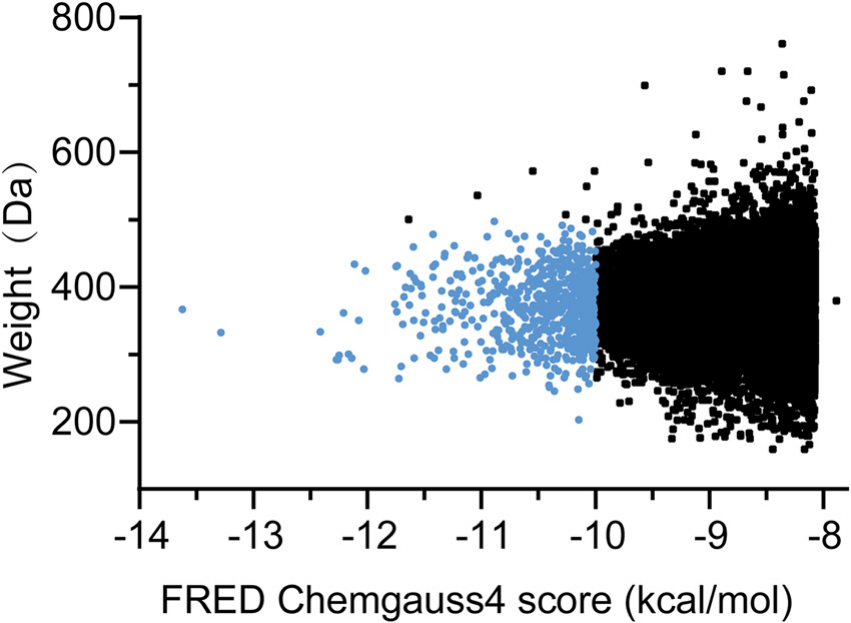
Binding energy and molecular weight for the top 30,000 compounds. The blue section comprises 682 compounds with molecular weights less than 500 Da and binding energies less than −10 kcal/mol.

A virtual screening was performed using the FRED software, where multiple conformations of each compound from the ChemDiv database (1,535,478 compounds, version 2019; ChemDiv, San Diego, CA) were docked to the LuxS pocket. The top 30,000 compounds with the best docking scores were retained, where the Chemgauss4 score function was applied. The binding energy and molecular weight distribution of the top 30,000 compounds are shown in Fig. 4. The results showed that the binding energy of the top 30,000 compounds was between −13.6 and −7.8 kcal/mol and that the molecular weight was between 100 and 800 Da. Most of these compounds have a binding energy between −10 and −7.8 kcal/mol, with 689 compounds having a binding energy greater than −10 kcal/mol; seven of these compounds with a molecular weight greater than 500 Da were deleted. Finally, 682 compounds with a molecular weight less than 500 Da and a binding energy less than −10 kcal/mol (indicated in blue in Fig. 4) were retained for subsequent analysis.

### Optimization of the docking mode for 682 compounds.

**FIG 5:**
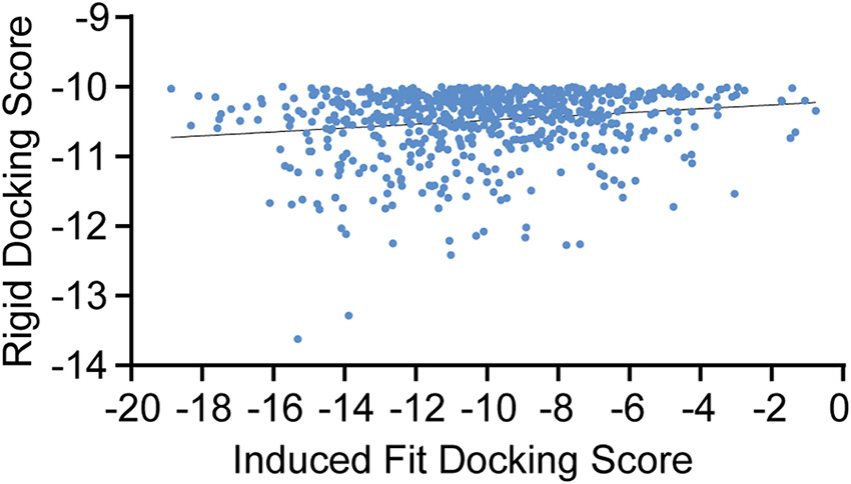
Correlation between optimized docking scores and rigid docking scores of 682 compounds.

Through the rigid docking strategy, 682 compounds with high affinity were screened from the ChemDiv database. The docking plug-in in MOE was then used to optimize the interaction between the 682 compounds and LuxS (Fig. 5). The docking score of the compounds was between −20 and 0 kcal/mol. There were 339 compounds with docking scores below −10 kcal/mol, a finding which had no significant correlation with the rigid docking score.

### Scaffold diversity and druggability analysis of 339 compounds.

**FIG 6:**
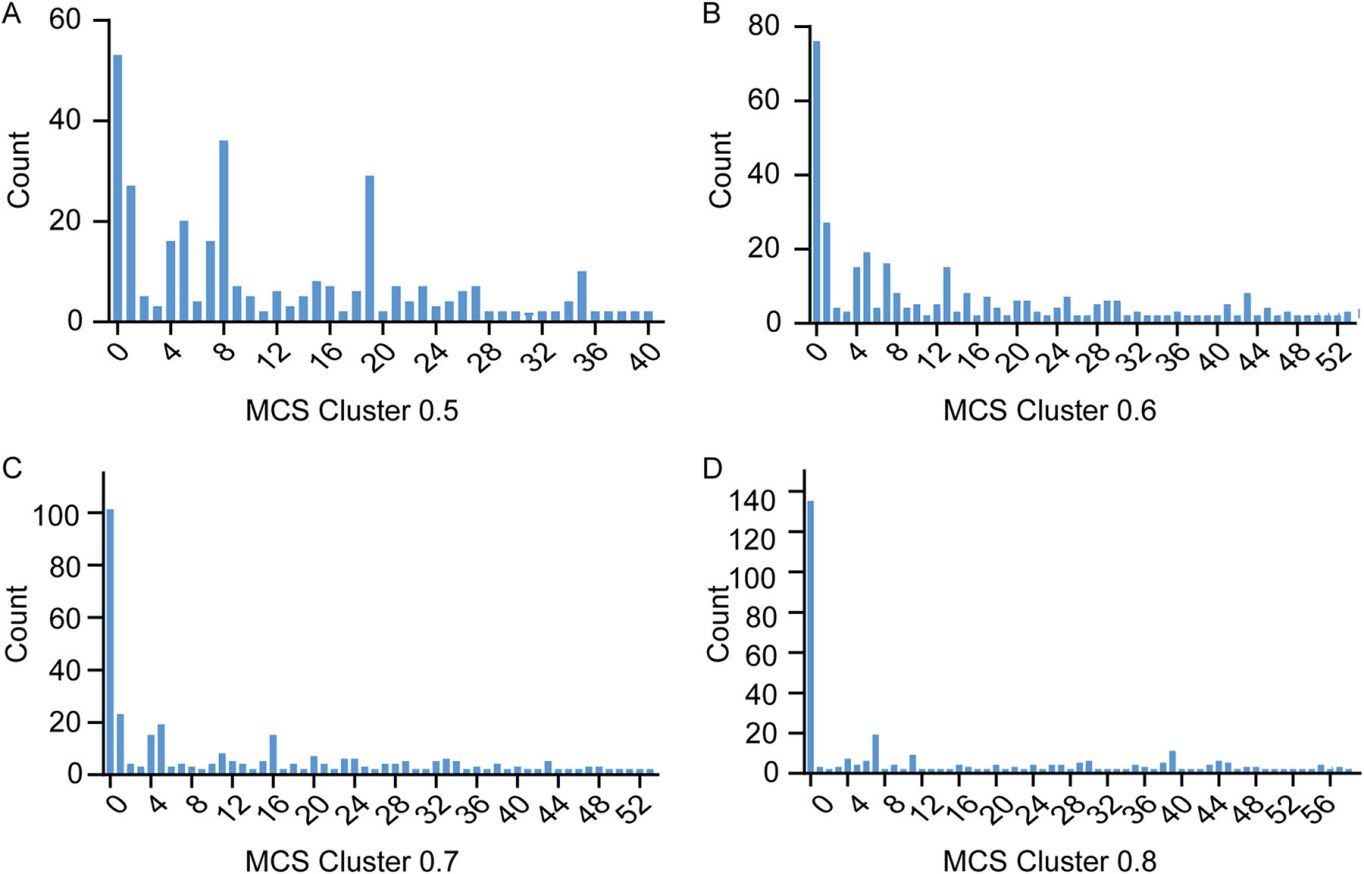
Clusters of 339 compounds based on the maximum common substructure (MCS), which uses a combination of 2D path-based fingerprints and a maximum common substructure algorithm. Compounds with common substructure are classified together. The compounds were clustered into 41 groups with a threshold of 0.5 (A), 53 groups with a threshold of 0.6 (B), 53 groups with a threshold of 0.7 (C), and 58 groups with a threshold of 0.8 (D).

The compounds with similar scaffolding were clustered together to analyze the structural diversity of the 339 compounds with binding energy lower than –10 kcal/mol. By adjusting the minimum similarity threshold to 0.5, 0.6, 0.7, and 0.8, the 339 compounds were clustered into 41, 53, 53, and 58 groups, respectively. Based on the minimum similarity threshold of 0.6, these compounds had maximum common substructure (MCS), and the number of compounds classified by each group was large (Fig. 6). Therefore, we selected the classification method with the minimum similarity threshold of 0.6 to analyze and screen the properties of these compounds.

**FIG 7:**
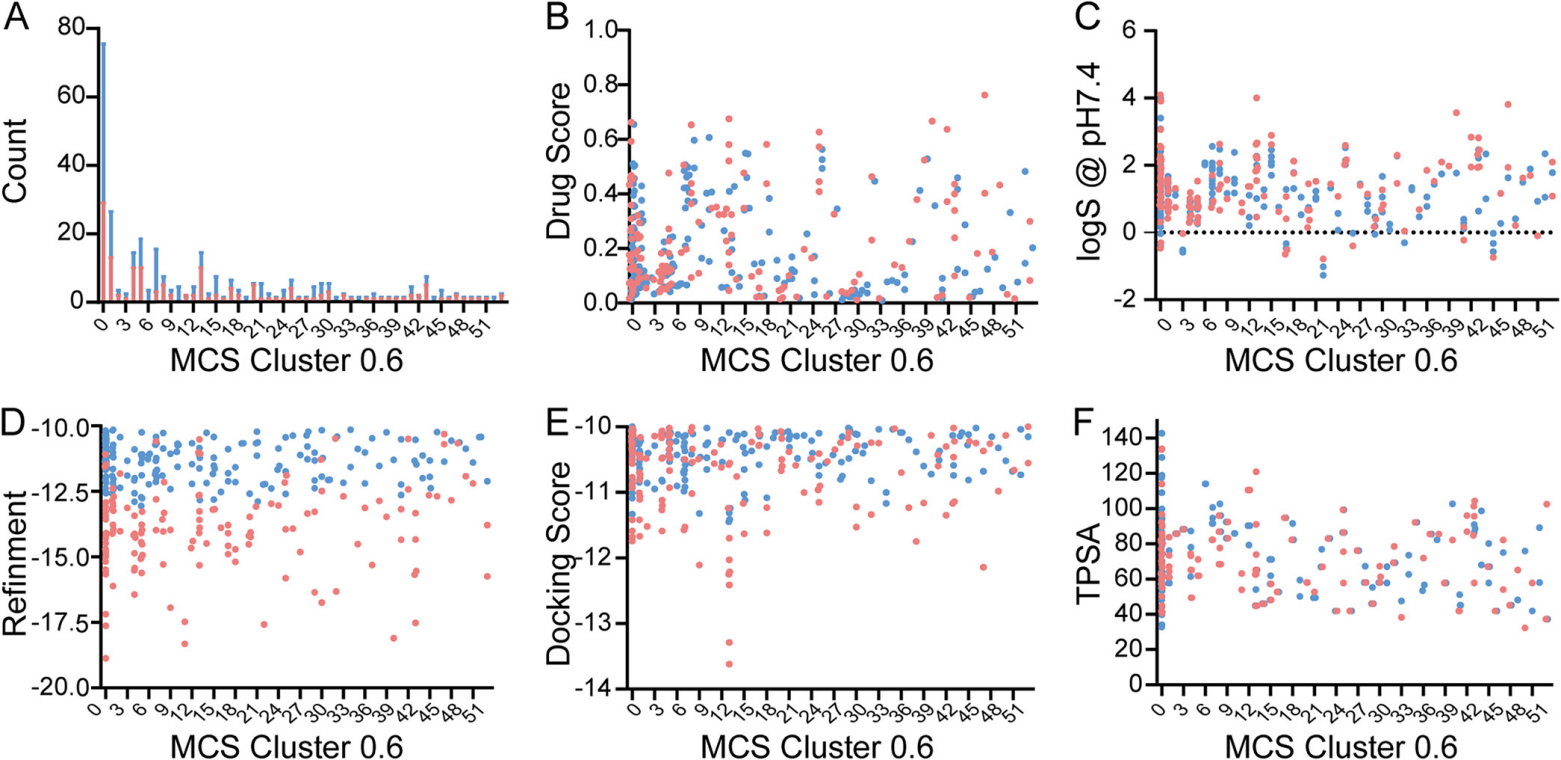
(A to F) Property distributions for count (A), drug score (B), *logS* at pH 7.4 (C), refinement (D), docking score (E), and TPSA (F) of the selected compounds in different groups (MCS with 0.6 thresholds). The 148 compounds with similar properties are indicated in red.

**FIG 8:**
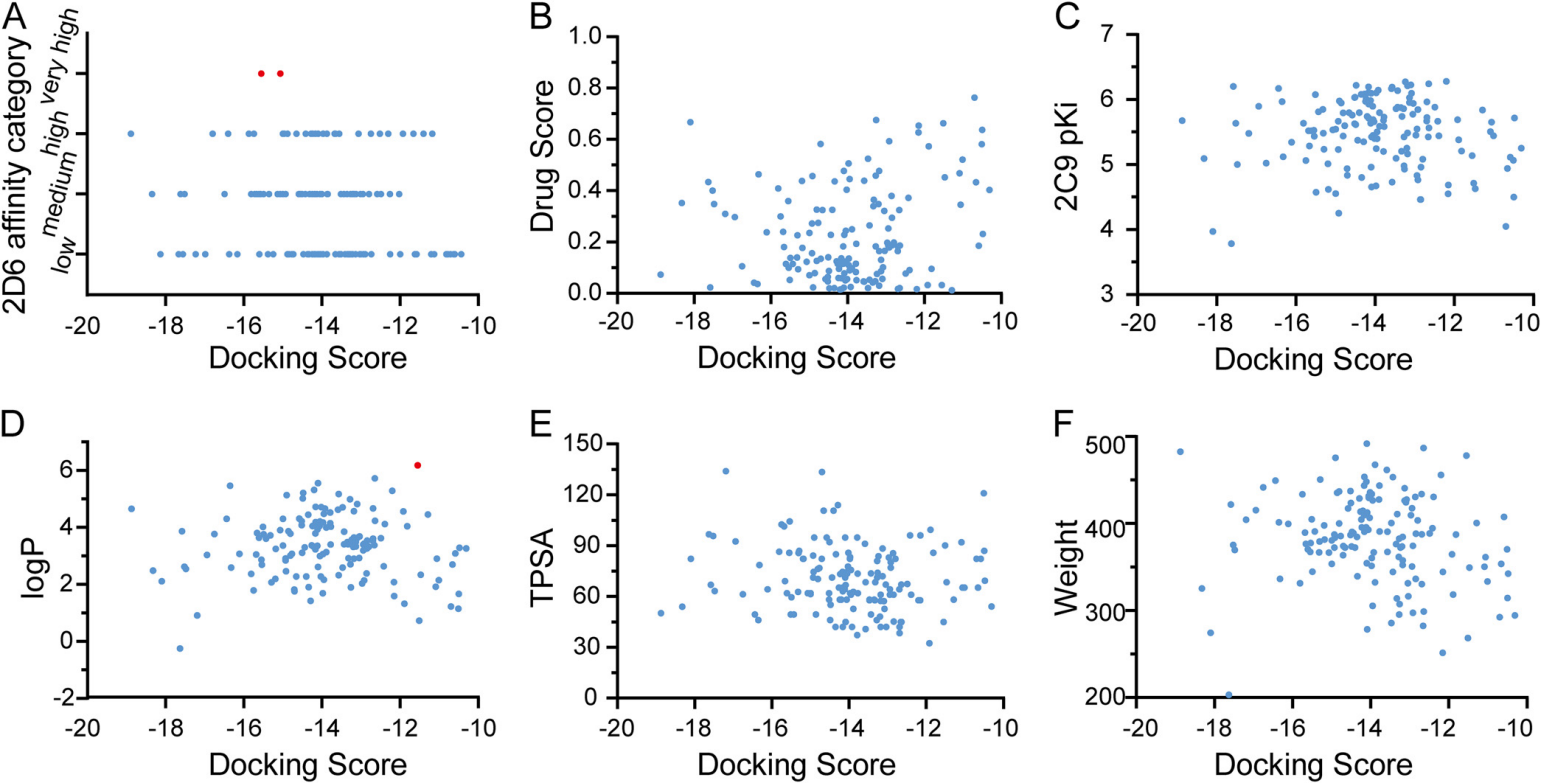
(A to F) Correlation between docking score and properties for 2D6 affinity category (A), drug score (B), 2C9 pKi (C), logP (D), TPSA (E), and weight (F) of 148 compounds.

Afterward, the StarDrop software (v6.5.0) was used to calculate and analyze the properties of the 399 compounds (Fig. 7). The druggability scores for the 339 compounds ranged from 0.009 to 0.77. Based on the common scaffold threshold of 0.6, 47 groups of compounds, including 148 compounds, were screened from 53 groups of compounds according to the non-central-nervous-system scoring profile. The 148 compounds had more than 46 common scaffolds, meeting the requirements for scaffold diversity. The docking scores of the 148 compounds ranged from −18.87 to −10.31 kcal/mol, the druggability scores ranged from 0.01 to 0.77, and the molecular weights ranged from 200 to 500 Da (Fig. 8). In addition, two compounds (8016-9608 and 8018-5506) had strong 2D6 enzyme affinity (very high; Fig. 8A, red dot); thus, they are very likely to be metabolized. Compound 8016-5502 could be metabolized by the 3A4 enzyme (logP > 6; Fig. 8D, red dot). Therefore, these three compounds were excluded, and 145 remaining compounds were selected for further study. The structure, properties, and diversity of the 145 selected compounds are shown in Table S1 in the supplemental material.

### Interaction mode between compounds and LuxS.

**FIG 9:**
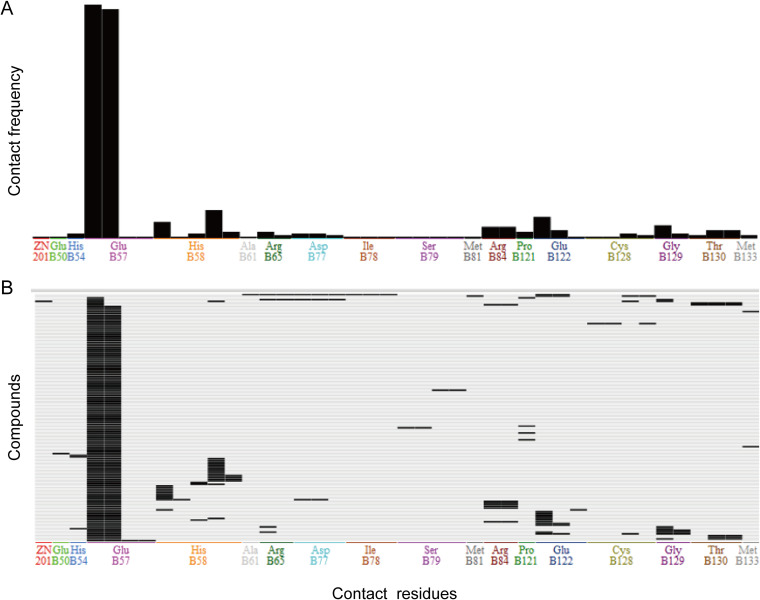
Interaction between 145 compounds and LuxS. (A) Contact frequency of amino acid residues binding to compounds. (B) Compounds that bound to amino acid residues.

Interaction modes between 145 compounds and the target protein (LuxS) are shown in Fig. 9. A total of 17 amino acid sites in the docking pocket participated in binding. Of these, amino acid E57 interacted with most compounds, except compounds 4470-0385 and G365-0523. Compound 8004-4282 bound with Zn^2+^, H58, C128, and G129. Twenty-three compounds interacted with Y495, 15 compounds bound with G496, and 14 compounds bound with R403. Among them, H54, H58, and C128 were the metal-binding sites of the LuxS enzyme. Compounds P132-0309, F692-0414, and F187-0533 interacted with amino acid H54 by π-π interaction. Compounds 8004-4282, D715-0786, 8017-9549, and G365-0523 interacted with site C128, while 29 compounds could directly interact with H58 through hydrogen bonds and π-π interaction. Based on the LuxS-methionine complex structure, the metal-binding sites were predicted as E57, H58, D77, I78, P80, and E122. Except for P80, other sites were involved in binding compounds, and E57 was the main binding site. Based on the binding mode, we found that all the screened compounds bound to methionine, and 35 compounds bound to metals. Therefore, it was speculated that these compounds could interfere with the activity of the LuxS enzyme.

### Screening of LuxS inhibitors against *E. coli*.

**FIG 10:**
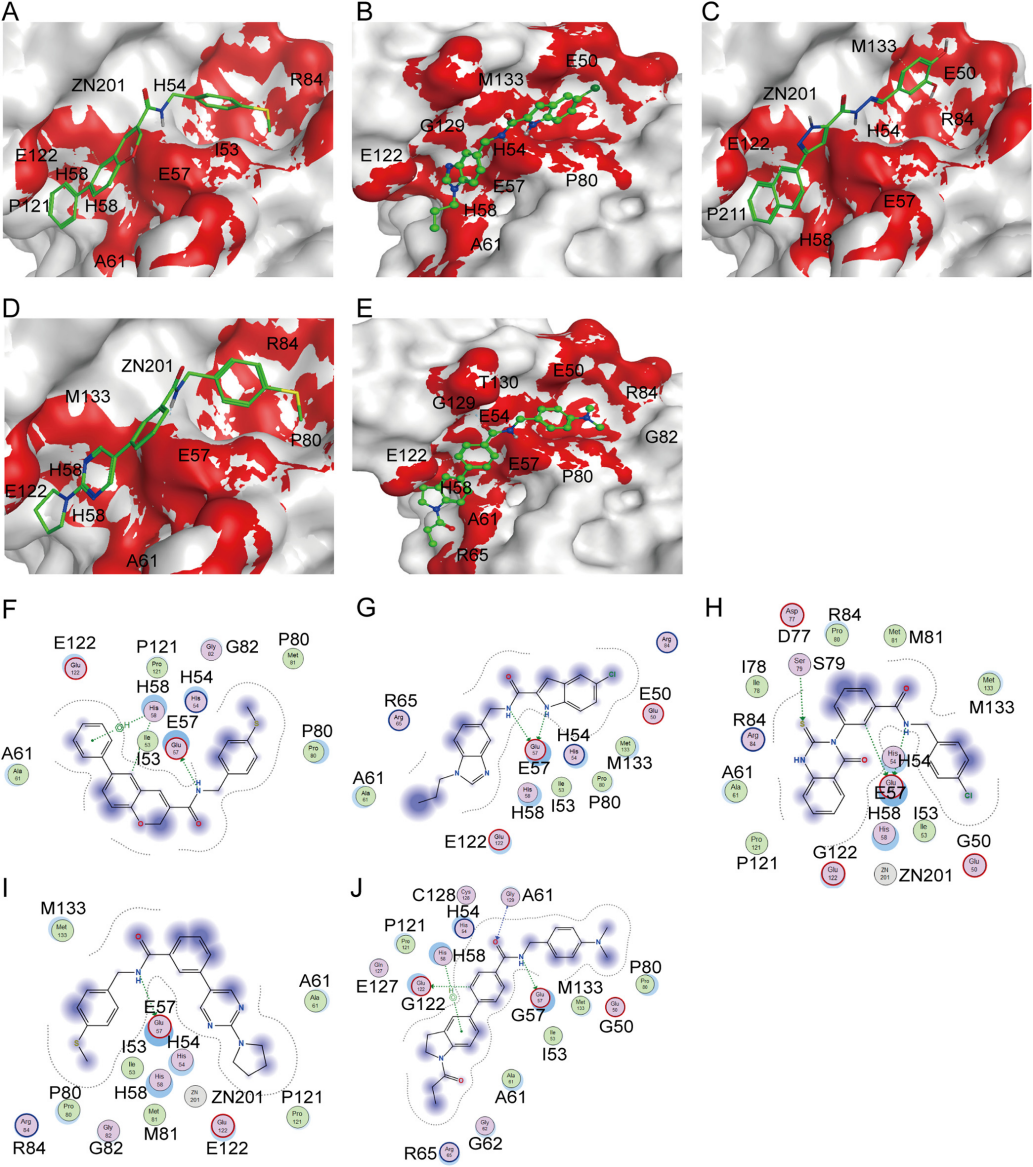
Interactions between five hit compounds and LuxS. (A to E) Spatial conformation and interaction amino acids of compounds L449-1159 (A), M414-3326 (B), 3254-3286 (C), L368-0079 (D), and L413-0180 (E) in the binding pocket. The red region indicates the binding region of small molecules. Compounds are shown in green spheres. (F to J) 2D interaction pattern diagram of compounds L449-1159 (F), M414-3326 (G), 3254-3286 (H), L368-0079 (I), and L413-0180 (J) with LuxS. Green dotted lines indicate hydrogen bonding. Blue dotted lines indicate the π-π stacking.

A total of 101 of 145 compounds were obtained from Shanghai TopScience Biotechnology Co., Ltd. (Shanghai, China). Inhibition of the 101 compounds on AI-2 production was assessed using the AI-2 bioluminescence method (see Table S3). Among them, the inhibition of 33 compounds on AI-2 production was higher than 65%. Furthermore, the half-inhibitory concentrations (IC_50_) of the 33 compounds were assessed using AI-2 bioluminescence assay (Table 1). The result showed that the IC_50_ values for five compounds (L449-1159, M414-3326, 3254-3286, L368-0079, and L413-0180) were <10 μM, so these five compounds were studied further. The interactions of the five hit compounds with LuxS are shown in Fig. 10. We found that hit compounds and LuxS interact through hydrogen bonding and π-π stacking. The docking scores of L449-1159, M414-3326, 3254-3286, L368-0079, and L413-0180 were −12.69, −15.32, −14.66, −14.24, and −13.08 kcal/mol, respectively. The L449-1159 compound formed two hydrogens bonds with amino acids E57 and H58 (Fig. 10A and F). The M414-3326 compound formed two hydrogen bond pairs with amino acid E57 (Fig. 10B and G). The 3254-3286 compound formed two hydrogens bonds and π-π stacking with amino acids E57 and M133 (Fig. 10C and H). The L368-0079 compound formed hydrogens bonds with amino acid E57 (Fig. 10D and I). Finally, the L413-0180 compound formed three hydrogens bonds with amino acids E57, H58, and E122, and π-π stacking with amino acid G129 (Fig. 10E and J).

**TABLE 1:** IC_50_ values for 33 compounds against LuxS of E. coli

No.	Compound	IC_50_ (μM)
1	L413-0180	4.028
2	L368-0079	7.006
3	3254-3286	7.573
4	L449-1159	8.639
5	M414-3326	9.617
6	G365-0523	12.21
7	F603-0744	12.32
8	Y040-2389	12.83
9	L413-0101	13.35
10	G945-0601	13.36
11	F187-0533	13.56
12	G883-0331	15.05
13	G751-0153	16.8
14	G883-0109	17.89
15	G883-0595	18.58
16	G883-0476	18.63
17	D753-0186	18.85
18	G883-0304	19.02
19	G883-0627	19.63
20	L010-0050	20.45
21	G883-0849	21.76
22	E983-1808	21.91
23	0180-0358	22.54
24	8004-4282	23.74
25	G751-5495	24.79
26	G883-2010	24.88
27	F379-1612	26.08
28	G751-5415	26.4
29	E941-0195	28.49
30	G883-0701	28.65
31	1761-1352	29.18
32	E950-0198	30.88
33	E950-0195	32.77

### ADMET properties of the hit compounds.

The ADMET properties of five hit compounds were predicated by the ADMET module of Discovery Studio 3.5. The solubility, blood-brain barrier (BBB), cytochrome p450 2D6-binding (CYP2D6), hepatotoxic, human intestinal absorption, and plasma protein binding (PPB) results of the compounds are shown in Table 2. The water solubility result showed that the solubility of the five hit compounds in water at 25°C was low. Except for 3254-3286, the rest of the compounds showed high blood-brain barrier permeability. The five hit compounds were predicted to have good intestinal absorption levels and plasma protein binding capacity, which indicates that the compounds have good cell permeability and plasma protein transport ability in human or animal. In addition, they did not inhibit the metabolic capacity of the metabolic enzyme (CYP2D6). In contrast, all compounds were hepatotoxic.

**TABLE 2:** ADME predictions for five hit compounds

Compound	Solubility level	BBB	CYP2D6	Hepatotoxic	Absorption level	PPB
L449-1159	1	0	False	True	0	True
M414-3326	1	1	False	True	0	True
3254-3286	2	4	False	True	1	True
L368-0079	2	1	False	True	0	True
L413-0180	2	1	False	True	0	True

Regarding toxicity, the ADMET module in Discovery Studio 3.5 was used to predict Ames mutagenicity, carcinogenicity (American National Toxicology Program [NTP]), ocular irritancy, and skin irritancy of the five compounds (Table 3). The results showed that the L449-1159 compound was not mutagenic and carcinogenic and could not cause eye and skin irritations. The L368-0079 and L413-0180 compounds were carcinogenic only to female rats but not to mice and male rats. In addition, they were nonmutagenic and could not cause oral and skin irritations. However, M414-3326 was mutagenic and carcinogenic to female rats and mice. Thus, structural modifications are needed to reduce their toxicity. Results were scored as follows: (i) solubility level—0 (extremely low), 1 (very low, but possible), 2 (low), or 3 (good); (ii) BBB level—0 (very high penetrant), 1 (high), 2 (medium), 3 (low), or 4 (undefined); cytochrome P450 2D6 level—false (noninhibitor) or true (inhibitor); hepatotoxicity—false (nontoxic) or true (toxic); absorption level—0 (good), 1 (moderate), 2 (poor), or 3 (very poor); and plasma protein binding—false (absorbent weak) or true (absorbent strong).

**TABLE 3:** Toxicity predictions for five hit compounds

Category	Compound toxicity prediction^*a*^				
	L449-1159	M414-3326	3254-3268	L368-0079	L413-0180
Ames_Mutagenicity	Nm	Mu	Mu	Nm	Nm
Mouse_Female_NTP	Nc	Ca	Nc	Nc	Nc
Mouse_Male_NTP	Nc	Nc	Nc	Nc	Nc
Ocular_Irritancy	Mild	Mild	Mild	Mild	Mild
Rat_Female_NTP	Nc	Ca	Nc	Ca	Ca
Rat_Male_NTP	Nc	Nc	Nc	Nc	Nc
Skin_Irritancy	Nc	Nc	Nc	Nc	Nc

a Mu, mutagen; Nm, nonmutagen; Ca, carcinogen; Nc, noncarcinogen.

### Molecular dynamics simulation.

**FIG 11:**
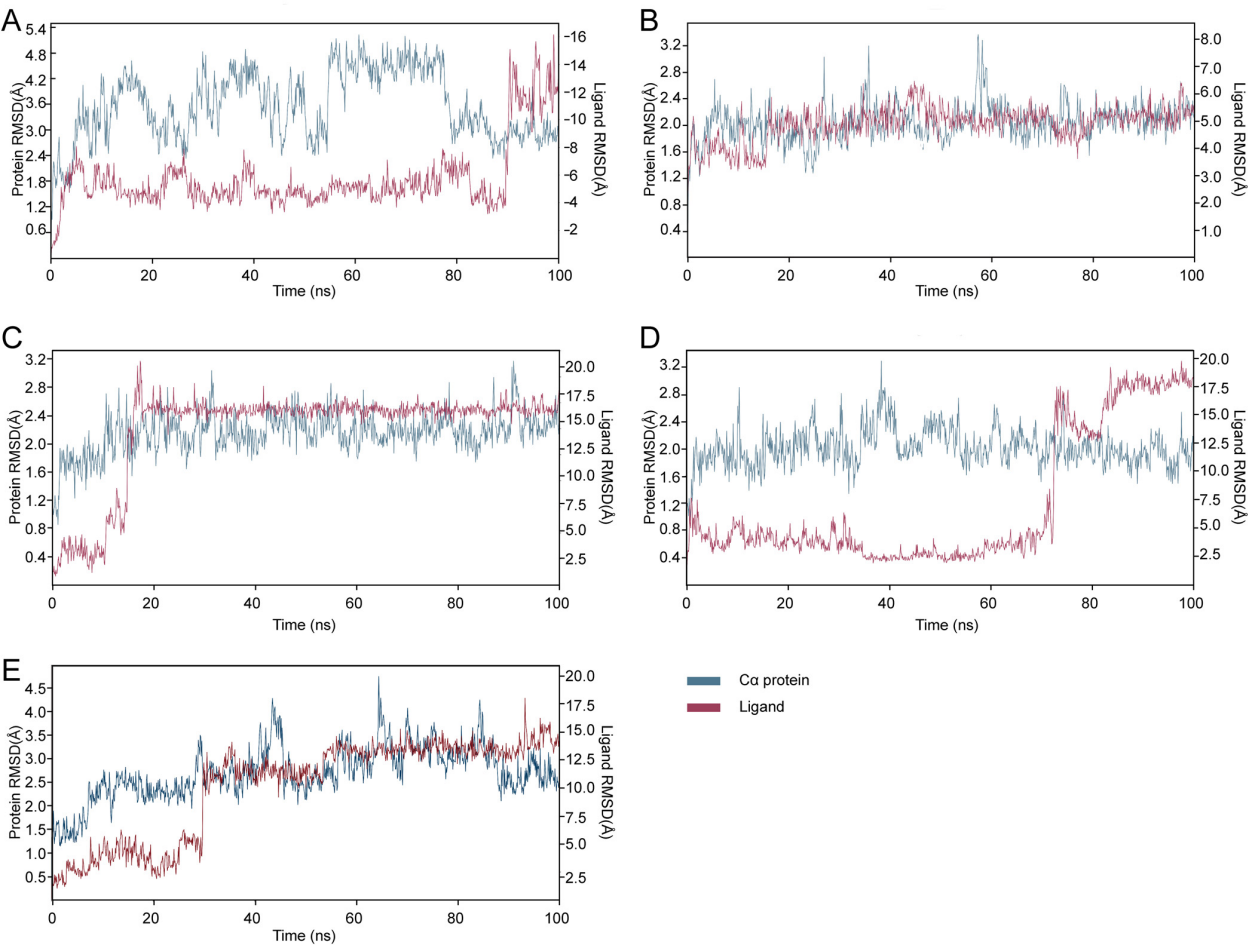
(A to E) Protein-ligand RMSD plot of compounds L449-1159 (A), M414-3326 (B), 3254-3286 (C), L368-0079 (D), and L413-0180 (E) binding to the LuxS protein. The analysis was performed using MD simulation.

Based on molecular docking, anti-QS activity screening, and ADMET prediction, molecular dynamics (MD) simulations of the five complexes were performed. Data from the Desmond software package are presented in the form of RMSD protein fluctuations, as shown in Fig. 11. Compounds M414-3326, 3254-3286, and L413-0180 were stable throughout the simulation process. The changes in RMSD were within the acceptable range (Fig. 11B, C, and E). Compound M414-3326 with LuxS complex was highly stable throughout the simulation, undergoing minimal conformational changes (Fig. 11B). The complexes of 3254-3286 and L413-0180 with LuxS were stable at 19 and 30 ns, respectively. However, the complexes of L449-1159 and L368-0079 with LuxS showed a sudden and significant fluctuation at ~76 ns, indicating that the binding of L449-1159 and L368-0079 with LuxS was unstable (Fig. 11A and D). Therefore, compounds M414-3326, 3254-3286, and L413-0180 were selected for further study.

### Surface plasmon resonance.

**FIG 12:**
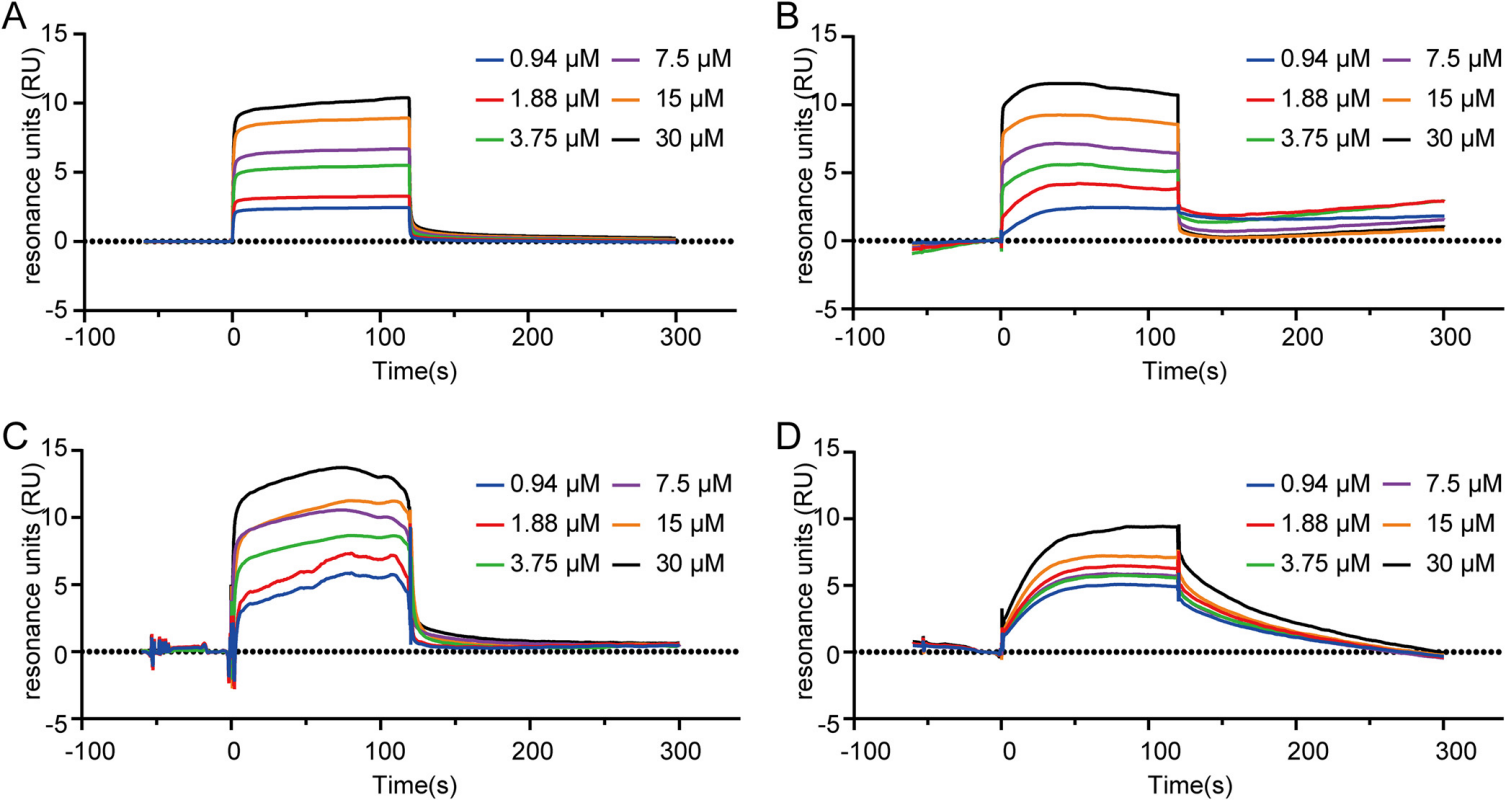
(A to D) Direct interaction between compounds furanone C30 (A), M414-3326 (B), 3254-3286 (C), and L413-0180 (D) with LuxS.

Surface plasmon resonance (SPR) was used to determine whether the three compounds could bind with LuxS and whether this contributed to their anti-QS activity. The results showed that the positive-control compound furanone C30 specifically bound to LuxS, and its equilibrium dissociation constant (*K_D_*) was 0.345 μM (Fig. 12A). In addition, the *K_D_* values for compounds M414-3326, 3254-3286, and L3368-0180 were 1.92, 18.5, and 77.7 μM, respectively (Fig. 12B to D). The results demonstrated that compounds M414-3326, 3254-3286, and L13-0180 could specifically bind to LuxS in a concentration-dependent manner.

### Effects of hit compounds on growth and metabolic activity of *E. coli* O157:H7.

**FIG 13:**
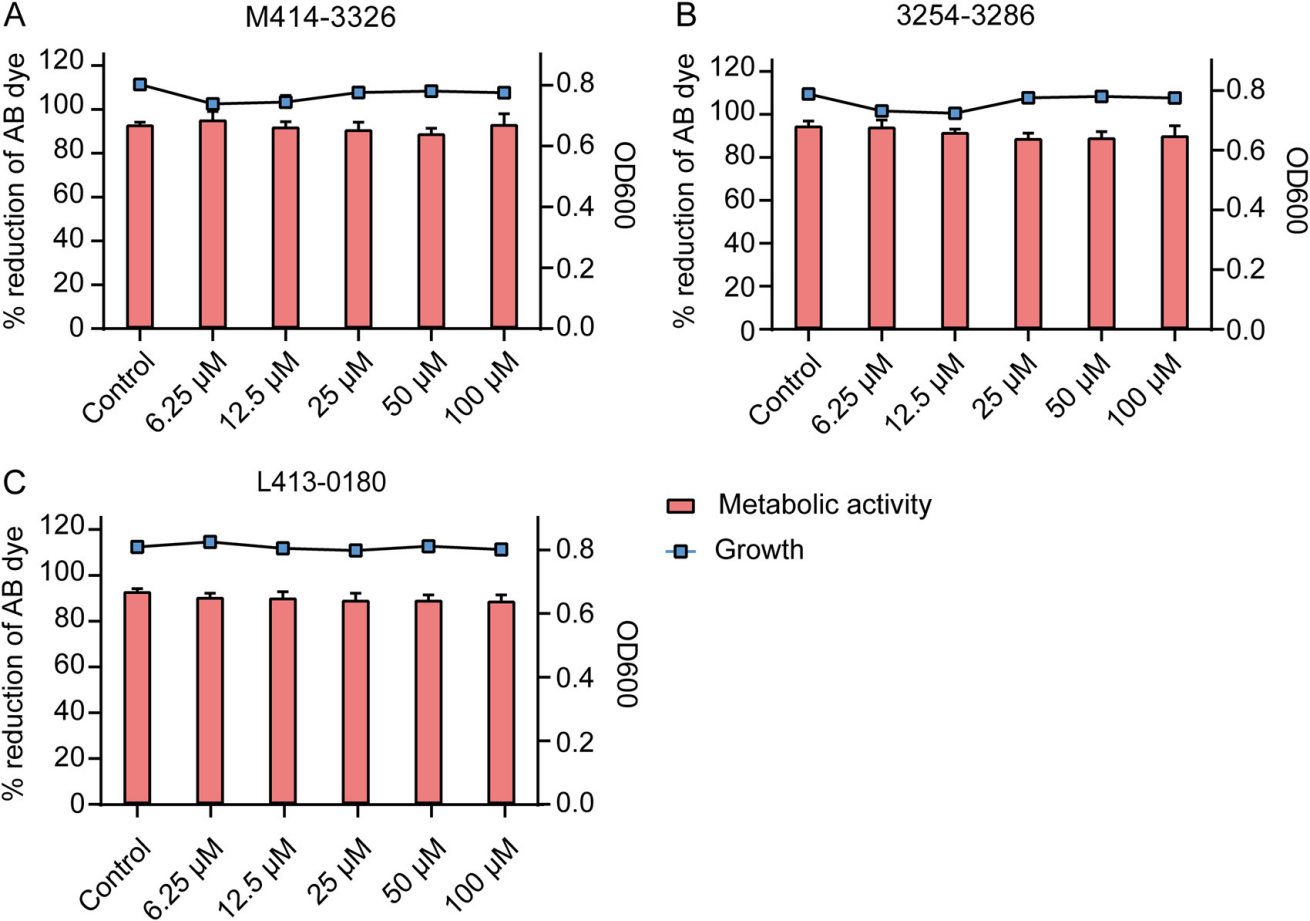
(A to C) Growth and metabolic activity of E. coli in the presence of hit compounds M414-3326 (A), 3254-3286 (B), and L368-0079 (C). The line graph shows the growth of E. coli in the presence of the hit compounds. The analysis was performed using a microbroth dilution assay. The bar graph shows the metabolic activity of E. coli based on the alamarBlue assay. Data represent means ± the standard deviations (SD) of three experiments conducted in triplicate.

The effects of three hit compounds (M414-3326, 3254-3286, and L413-0180) on the growth and metabolism of E. coli were determined using microbroth dilution and alamarBlue assays. The results showed that in the concentration range of 6.25 to 100 μM, the three hit compounds had no significant effect on the growth and metabolic activity of E. coli compared to the control (Fig. 13). These results confirmed the nonantibacterial effect of the three hit compounds.

### Cytotoxicity of hit compounds to Caco-2.

**FIG 14:**
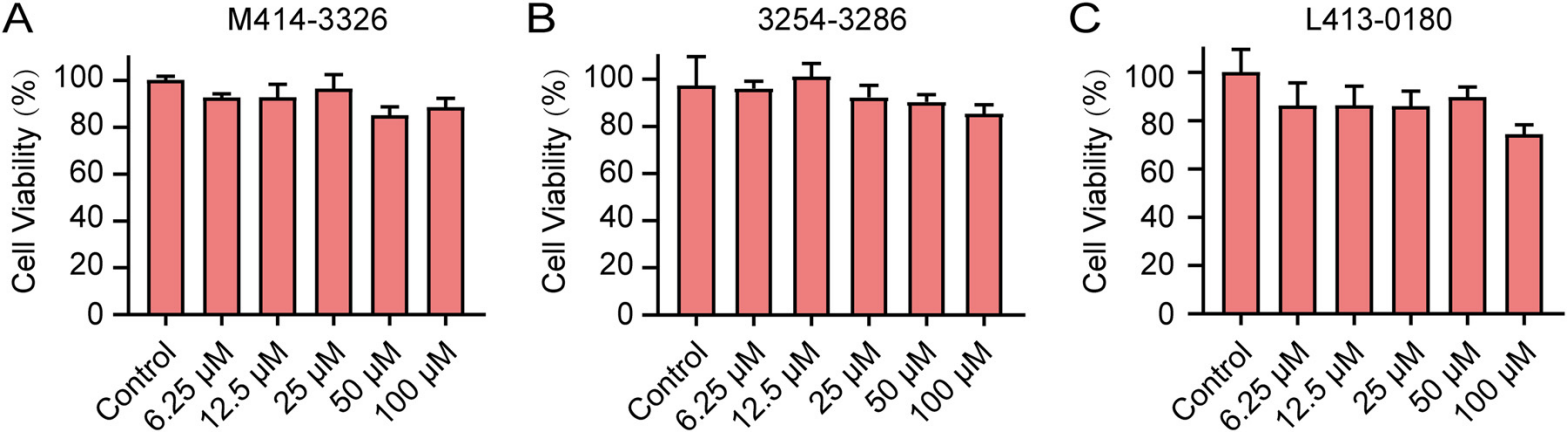
(A to C) Cytotoxicity of the three hit compounds M414-3326 (A), 3254-3286 (B), and L413-0180 (C) to Caco-2. The cells were treated with hit compounds at different concentrations (0, 6.25, 12.5, 25, 50, and 100 μM) for 24 h. Data represent means ± the SD of three experiments conducted in triplicate.

The cytotoxicity of the three hit compounds to Caco-2 cells was determined using the CCK-8 assay. The results showed that hit compounds M414-3326 and 3254–3286 were nontoxic to Caco-2 cells in the concentration range of 6.25 to 100 μM. Compound L413-0180 was slightly cytotoxic at 100 μM but nontoxic at 6.25 to 50 μM (Fig. 14).

### Effects of hit compounds on biofilm formation of *E. coli*.

**FIG 15:**
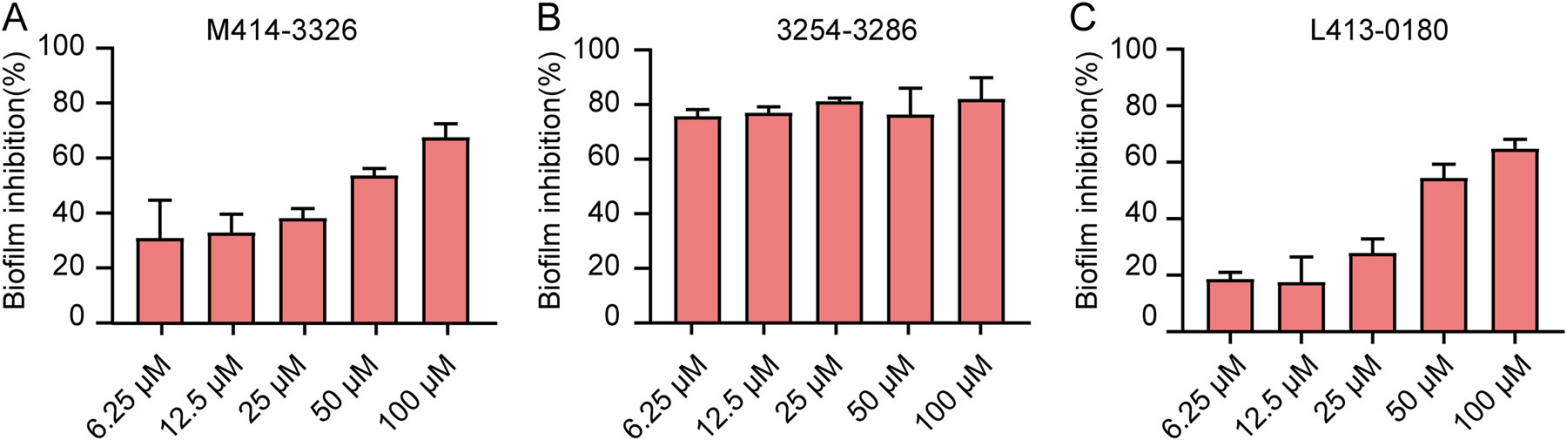
(A to C) Effect of the three hit compounds M414-3326 (A), 3254-3286 (B), and L413-0180 (C) on E. coli biofilm formation. Inhibition of biofilm formation at various concentrations of the three hit compounds (6.25, 12.5, 25, 50, and 100 μM) was determined for 24 h through CV staining. Data represent means ± the SD of three experiments performed in triplicate.

**FIG 16:**
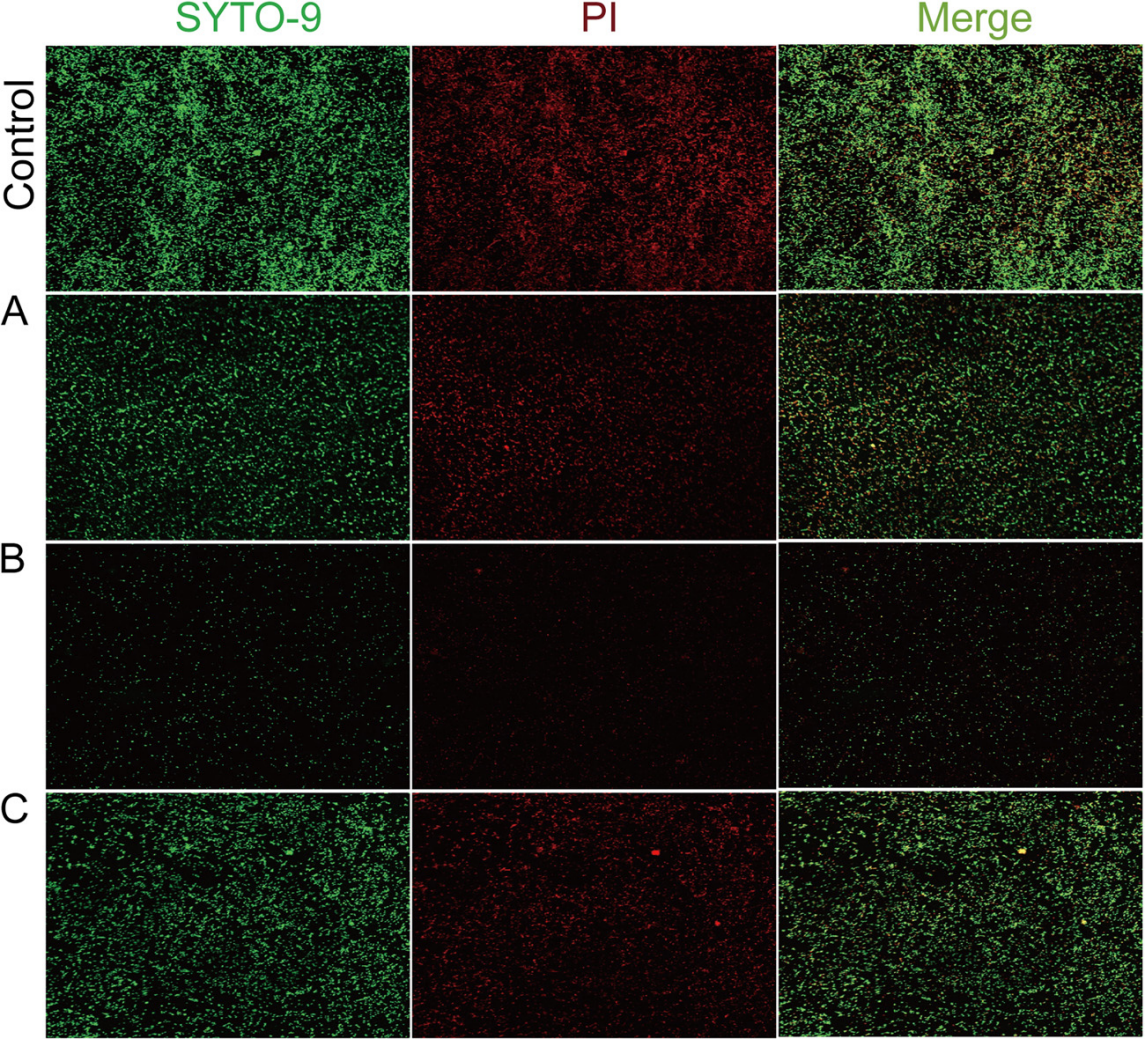
CLSM images of E. coli biofilm formation. Biofilms with three hit compounds (50 μM) for 24 h were examined using CLSM. (A) M414-3326; (B) 3254-3286; (C) L413-0180.

The biofilm inhibition of the three hit compounds was measured through crystal violet (CV) staining. The results showed that the three hit compounds significantly reduced biofilm formation at concentrations 6.25, 12.5, 25, 50, and 100 μM (Fig. 15). Compounds L449-1159, M414-3326, 3254-3286, L368-0079, and L413-0180 increased the biofilm formation by about 75.4, 53.6, 81.12, 76.8, and 51.4%, respectively, at 50 μM for 24 h. In addition, confocal laser scanning microscopy (CLSM) image analysis was performed using the fluorescent dye SYTO9 and propidium iodide (PI) to evaluate whether the compounds inhibited biofilm formation (Fig. 16). The result showed that hit compounds significantly inhibited biofilm formation, as demonstrated by reducing the green fluorescence (live cells) and red fluorescence (dead cells) of E. coli cells. Generally, the results demonstrated that the three hit compounds inhibited biofilm formation.

### Effect of hit compounds on the expression of QS-regulated genes of *E. coli*.

**FIG 17:**
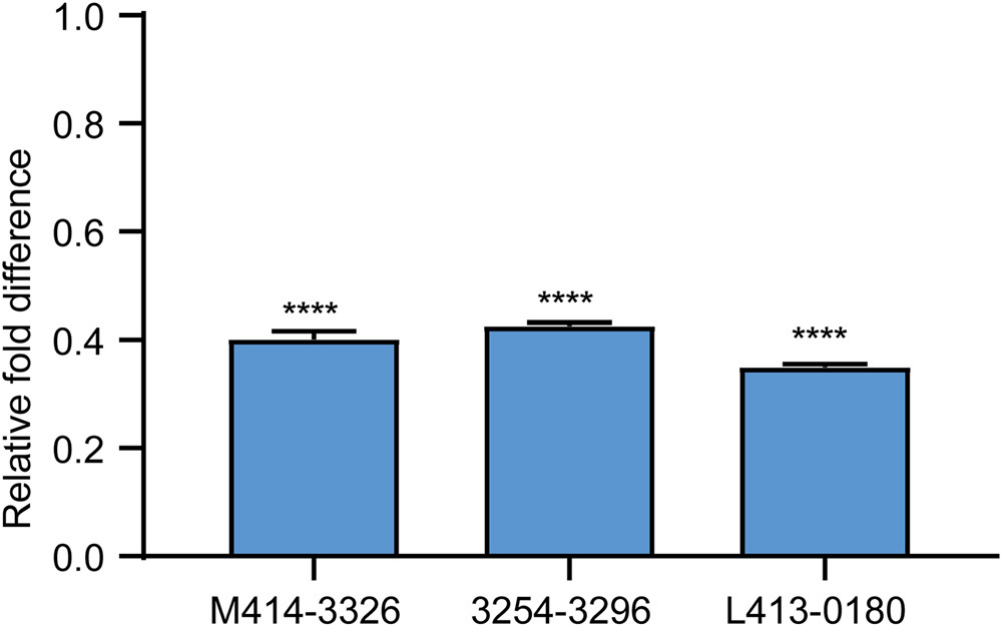
Effect of the three hit compounds on expression of the *luxS* gene. The results revealed that all hit compounds downregulated the expression of luxS. The results from all experiments are presented as the means ± the SD of three replicates (****, *P < *0.001).

The quantitative reverse transcription-PCR (qRT-PCR) was used to assess the effect of the three hit compounds (50 μM) on the expression of the LuxS of E. coli. Compounds M414-3326, 3254-3286, and L413-0180 suppressed the expression of LuxS by about 59.9, 57.4, and 65.1%, respectively, at 50 μM for 24 h (Fig. 17). The result demonstrated that compounds M414-3326, 3254-3286, and L413-0180 inhibited the activity of the LuxS enzyme.

Due to the massive use and abuse of antibiotics, antibiotic resistance has become a major threat to public health and economic development (47). Traditional antibacterial drugs usually interfere with the efflux pump system, DNA replication, protein synthesis, or bacterial cell wall biosynthesis (48–50). However, due to the abuse of antibiotics in past decades, evolutionary pressure forced bacteria to develop strategies to overcome the effects of antibiotics, which led to the emergence of drug resistance (51). Therefore, new targets are needed to treat these bacterial infections. The QS system is a bacterial communication mechanism that can regulate the formation of bacterial resistance (28, 29), biofilm formation (26, 27), and virulence factors (22, 23, 52). In addition, biofilms formed by many pathogens also lead to strong bacterial resistance (53). The QS system can reduce antibiotic resistance by regulating biofilm formation (50). Therefore, QS inhibition potentially forms a new antibacterial strategy to prevent bacterial resistance and inhibit the production of virulence factors related to population density.

In addition, these virulence factors are not necessary for the growth and survival of bacteria. Therefore, centralized treatment will not inhibit the growth of bacteria and generate selective pressure. It can effectively reduce the development of bacterial resistance (30–32). The progress of QS research could lead to the development of new antibacterial compounds with new modes of action, possibly providing a new strategy against antimicrobial resistance. The bacterial LuxS protein plays a key role in the QS process of E. coli and is an effective target (54). In the present study, the LuxS protein was used as a target. Three new structural inhibitors inhibiting QS that were different from previously reported structures were obtained through virtual screening technology (42–44). The three compounds—M414-3326, 3254-3286, and L413-0180—could specifically and stably combine with LuxS protein. Moreover, these compounds inhibited biofilm formation without adverse effects on bacterial growth and metabolism. In conclusion, the three compounds can be used as QSIs or lead compounds in the future.

### Conclusions.

In this study, QS inhibitors with high affinity to the target protein (LuxS) of E. coli were obtained through virtual screening. Five hit compounds—L449-1159, M414-3326, 3254-3286, L368-0079, and L413-0180—inhibiting the production of type II QS signal molecule AI-2 were also screened using the AI-2 bioluminescence method. The ADMET properties predicted that these five compounds had good intestinal absorption levels and plasma protein binding and did not inhibit the metabolism of the CYP2D6 metabolic enzyme. In addition, through molecular dynamics simulation and SPR experiments, we found that three of the five compounds (M414-3326, 3254-3286, and L413-0180) were stable and could specifically bind to LuxS. Further analysis showed that the three hit compounds significantly inhibit the biofilm formation of E. coli through CV staining and CLSM. Moreover, qRT-PCR results demonstrated that the compounds downregulated the expression of the LuxS gene. In summary, these results indicate that five hit compounds have antibiofilm and anti-QS properties. Thus, they could be used for treating E. coli O157:H7 infection.

## MATERIALS AND METHODS

### Bacterial strains and reagents.

E. coli O157:H7 (ATCC 43895) was purchased from Beina Chuanglian Biotechnology Research Institute (Beijing, China). Luria-Bertani broth (HuanKai Microbial, Guangdong, China) and Luria-Bertani agar (HuanKai Microbial) medium were used to cultivate E. coli. The V. harveyi strains BB170 and BB152 were provided in kind by Han Xiangan, a researcher at the Shanghai Veterinary Research Institute, Chinese Academy of Agricultural Sciences. Autoinducer bioassay (AB) medium was supplemented with 1 mM l-arginine, 1% glycerol, and 10 mM phosphate buffer (pH 7.2) for culturing V. harveyi.

The ChemDiv database, a commercially available small-molecule database from Shanghai TopScience Biotechnology Co. with more than 1 million compounds, was used as the screening library. A total of 101 selected compounds were purchased from Shanghai TopScience Biotechnology Co., Ltd.

### Homology modeling and model evaluation.

Using chain B in the high-resolution crystal structure of LuxS-quorum sensor molecular complex from Salmonella enterica serovar Typhi (PDB 5E68) as a template, a 3D structure model of LuxS of E. coli was developed using SWISS-MODEL (55). The model quality was assessed by qualitative model energy analysis (QMEAN) based on statistical methods and energy analysis (56).

### Prediction of small-molecule binding pockets.

The MOE software was used to analyze and optimize the homology modeling results (57). The process involves removing irrelevant molecules and protonation hydrogenation. Subsequently, the SiteFinder module of MOE software was used to analyze the small-molecule binding pockets on its surface.

### Virtual screening. (i) Preparation of protein receptor.

The 3D model of LuxS was generated into a receptor file by protonation and structural optimization. We used MOE SiteFinder software to redefine the binding pocket on the LuxS as the docking area for virtual screening (57). The apopdb2 of the receptor tool of OpenEye (release 3.2.0.2) was used to process and generate virtual screening receptor files in which the screening box was a docking box of 21.33 Å × 26.50 Å × 18.67 Å with a volume of 10,552 Å^3^.

### (ii) Preparation of small-molecule compound library.

The ChemDiv (version 2019) database, with up to 1,535,478 compounds, was selected as a virtual screening library of compounds. To ensure the global conformation of small molecules in the virtual screening process, the omega2 module (v3.0.1.2) in OpenEye software was employed to produce small-molecule conformations (57).

### Molecular docking score.

The virtual screening was performed against LuxS using the FRED software (v3.2.0.2) (58). The operating parameters (including save_component_scores) were selected as true, hitlist_size was selected as 30,000, docked_molecule_file was selected as sdf format, and other parameters were adopted as default parameters. The binding energy score was also used to assess docking conformations. Compounds with a binding energy of less than −10 kcal/mol and molecular weights of <500 were selected for subsequent analysis. In addition, the docking plug-in in the MOE software was used to optimize the interaction mode of small-molecule compounds (57). Moreover, cluster analysis of compounds with similar scaffolds was performed following the common substructure, and the druggability of each small molecule was scored by the StarDrop software (v6.5.0) using a non-central-nervous-system scoring profile (59). The scoring criteria for druggability included the following: logS (water solubility) > 1; HIA (human intestinal absorption) category, +; logP (octanol-water partition coefficients), >3.5; hERG (the human Ether-à-go-go-Related Gene), pIC50 ≤ 5; 2D6 (cytochrome P450 2D6) affinity category, low medium; 2C9 (cytochrome P450 2C9), pKi ≤ 6; P-gp (P-glycoprotein) category, no; PPB90 (plasma protein binding) category, low; and BBB, log(brain:blood), ≤−0.5. Finally, the protein-ligand interface fingerprinting method was used to analyze the interaction sites and types of forces between them and the target-receptor binding domain.

### Screening of anti-QS inhibitors.

An AI-2 bioluminescence assay with minor modifications was used as described previously to further assess the mechanisms of the inhibitors (60, 61). Briefly, E. coli O157:H7 was cultured for 16 h with 50 μM inhibitors. Cell-free supernatant was collected in a 0.22-μm-pore-size filter after centrifugation at 12,000 × *g* for 5 min. The V. harveyi BB170 grew in AB medium to an optical density at 600 nm (OD_600_) of 1.0 to 1.1 at 30°C under shaking incubation before being diluted at 1:2,500 with fresh AB medium. Exactly 20 μL of cell-free supernatant mixed with 180 μL of V. harveyi BB170 culture was transferred to black 96-well plates (Jingan, Shanghai, China) and incubated at 30°C in the dark for 3.5 h. The bioluminescence was measured using a multipurpose microplate reader (Enspire; Perkin-Elmer, USA). Cell-free supernatants of V. harveyi BB152 overnight cultures were used as the control. Further analysis was performed on the compound with the highest AI-2 inhibition. IC_50_ values were established using GraphPad Prism Software.

### Prediction of pharmacokinetic parameters and toxicological properties.

Pharmacokinetic parameters and toxicological properties include absorption, distribution, metabolism, excretion, and toxicity (ADMET) of molecules in organisms. The prediction of ADMET properties of drugs can effectively guide structural optimization, improve the success rate of drug development, and reduce its cost. To further assess the druggability of five hit compounds, the ADMET module of Discovery Studio (v3.5) was used to predict their ADMET properties (62).

### Molecular dynamics simulation.

Molecular dynamics of five complexes were conducted to optimize the conformation and evaluate the binding mode by Desmond suit (Academic Edition) of Schrödinger LLC (63). The simulation systems were prepared by applying the OPLS4 force field to the complex; an orthorhombic box was generated by a 10-Å border from the complexes. Then, SPC water molecules were used to fill the box, whereas 1.5 M NaCl and additional Na^+^ and Cl^–^ were also added to randomly neutralize the system (the other parameters were set up by default). The MD simulation total time was 100 ns. The electrostatic interaction was calculated using the Eswald method.

### SPR analysis.

The binding affinity of the three compounds and LuxS was assessed by SPR assay using a BIAcore T200 (GE Healthcare, Pittsburgh, PA) (64). The LuxS protein was immobilized on a CM5 sensor chip with the final immobilization levels of 7,582.4 resonance units (RU). The running buffer used in the study was 1.0× phosphate-buffered saline containing detergent (PBS-P^+^; pH 7.4) and 5% (vol/vol) dimethyl sulfoxide. For each binding cycle, the compound was injected for 120 s with a dissociation time of 180 s. A series of compound concentrations (0.94, 1.88, 3.75, 7.5, 15, and 30 μM) were injected for analysis, with furanone C30 as a positive control. Eventually, data were analyzed by Biacore evaluation software, whereas the equilibrium dissociation constant (*K_D_* = *K_d_*/*K_a_*) was calculated by curve fitting with a 1:1 binding model.

### Growth and metabolic activity.

As described for previous protocols, growth and metabolic activity were evaluated by the broth dilution method and an alamarBlue assay (65, 66). Briefly, 96-well plates were seeded with bacterial suspension (OD_600_ = 0.01) containing different concentrations of hit compounds and incubated at 37°C for 24 h. At 600 nm, the absorbance was measured using the Multiskan Go Reader (Thermo Fisher Scientific, USA).

Furthermore, the alamarBlue assay was performed to detect the metabolic activity of hit compounds (66). The bacterial suspension (OD_600_ = 0.01) with different concentrations of hit compounds was incubated at 37°C for 24 h. Cells from each well were harvested at 10,000 × *g* for 5 min, followed by two washes with phosphate-buffered saline (PBS; pH 7.2). The bacterial precipitation was resuspended with 90 μL of PBS and incubated with 10 μL of alamarBlue (Invitrogen; Thermo Fisher Scientific) at 37°C for 1 h. Of note, PBS containing only AB dye was considered a blank. The metabolic activity was computed based on the absorbance at 570 and 600 nm according to the following formula:

$$\text{metabolic activity (\%) }=\frac{\left(E_{\mathrm{oxi}(\text{OD570})}\times T_{\text{OD570}})\;-\;(E_{\mathrm{oxi}(\text{OD600})}\times T_{\text{OD600}}\right)}{\left(E_{\mathrm{red}(\text{OD570})}\times B_{\text{OD570}})\;-\;(E_{\mathrm{red}(\text{OD600})}\times B_{\text{OD600}}\right)}\times 100\%$$

where *E_oxi_*_(OD570)_ is the extinction coefficient in the oxidized form of AB at 570 nm = 80,586, *E_red_*_(OD570)_ is the extinction coefficient in the reduced form of AB at 570 nm = 155,677, *E_oxi_*_(OD600)_ is the extinction coefficient in the oxidized form of AB at 600 nm = 117,216, and *E_red_*_(OD600)_ is the extinction coefficient in the reduced form of AB at 570 nm = 14,652. *B* represents blanks, and *T* represents samples.

### Cytotoxicity.

The cytotoxicity of hit compounds was assessed by a CCK-8 assay in Caco-2 cells. At 37°C, Caco-2 cells (1 × 10^5^ cells/mL) were seeded in a 96-well plate for 24 h. Further, different concentrations of hit compounds were added to 96-well plates at final concentrations of 6.25, 12.5, 25, 50, and 100 μM. After incubation for 24 h, the plate was incubated at 37°C for 1 h with 10 μL of CCK-8 (MCE, China). A Multiskan Go Reader (Thermo Fisher Scientific) was used to measure the absorbance at 450 nm.

### Biofilm assay. (i) CV staining.

Based on previous studies, CV staining was used to assess biofilm formation (67). The overnight cultured E. coli O157:H7 was diluted to OD_600_ of 0.01. The diluted E. coli suspensions were mixed with different concentrations of hit compounds and incubated at 37°C for 24 h. The plate was washed three times using PBS (pH 7.2) before it was fixed with methanol for 30 min. After staining for 30 min with CV, the plates were rinsed with distilled water and dried at 55°C. Finally, the CV attached to wells was dissolved in 95% ethanol. The absorbance at 570 nm was then measured using a Multiskan Go Reader.

### (ii) CLSM.

The 3D image of E. coli biofilm was determined using CLSM based on protocols from the previous study with slight modifications (68). Briefly, bacterial suspensions (OD_600_ = 0.01) with different concentrations of hit compounds were seeded into 6-well plates with coverslips, followed by incubation at 37°C for 24 h. After the wells were washed with PBS (pH 7.2), the biofilm was stained using BacLight Live/Dead viability kit (Invitrogen L7012; Thermo Fisher Scientific) according to a standard procedure. Two stock solutions of SYTO9 and PI were diluted in PBS (pH 7.2), and 500 μL was added to each well at a 1 mL/3 μL/3 μL ratio. The washing holes were dried after 15 min. Live cells stained with SYTO9 and dead cells stained with PI were observed by CLSM (LSM800; Zeiss, Tokyo, Japan).

### (iii) qRT-PCR.

qRT-PCR was used to measure the LuxS expression in hit compounds. E. coli was incubated in the 12-well plate with or without hit compounds at 37°C for 24 h. A bacterial RNA kit (Omega, USA) was used to extract the total RNA. RNA quantity was measured using a NanoDrop OneC spectrophotometer (Thermo Scientific). A PrimeScript RT reagent kit with gDNA Eraser (TaKaRa Corporation, Japan) was used to reverse transcribe RNA into cDNA. qRT-PCR results were analyzed using TB Green Premix *Ex Taq* II (Tli RNase H Plus; TaKaRa Corporation). The relative changes in gene expression levels were analyzed based on the 2^–ΔΔ^*^CT^* method. The *gapA* gene was used as an internal control (69). Table S1 lists the primers used in this study.

### Statistical analysis.

Group comparisons were performed using nonparametric one-way analysis of variance in Prism 8 (GraphPad). Significance results are indicated in the figures, where applicable, by asterisks (*, *P < *0.05; ** *P < *0.01; ***, *P < *0.001; ****, *P < *0.0001).
