# Supplementary material for: Virtual Screening and In Vitro Experimental Verification of LuxS Inhibitors for Escherichia coli O157:H7
Source: Microbiol Spectr. 2023 Feb 21;11(2):e03502-22. doi: 10.1128/spectrum.03502-22 (PMC10100900; doi:10.1128/spectrum.03502-22)
Supplement: Supplemental file 1 — Supplemental material. Download spectrum.03502-22-s0001.pdf, PDF file, 1.7 MB [file spectrum.03502-22-s0001.pdf]

Virtual Screening and in Vitro Experimental Verification of LuxS Inhibitors for Escherichia coli O157:H7

Yu-Bin Bai, [a,b,c] Xiao-Rong Yang, [a,b,c] Bing Li, [a,b,c] Xu-Zheng Zhou, [a,b,c] Wei-Wei Wang, [a,b,c] Fu-Sheng Cheng, [a,b,c] and Ji-Yu Zhang [*a,b,c]

[a] A Key Laboratory of New Animal Drug Project of Gansu Province, Lanzhou, Gansu Province 730050, People's Republic of China.

[b] Key Laboratory of Veterinary Pharmaceutical Development, Ministry of Agriculture, Lanzhou, Gansu Province 730050, People's Republic of China.

[c] Lanzhou Institute of Husbandry and Pharmaceutical Sciences, Chinese Academy of Agricultural Sciences, Lanzhou, Gansu Province 730050, People's Republic of China

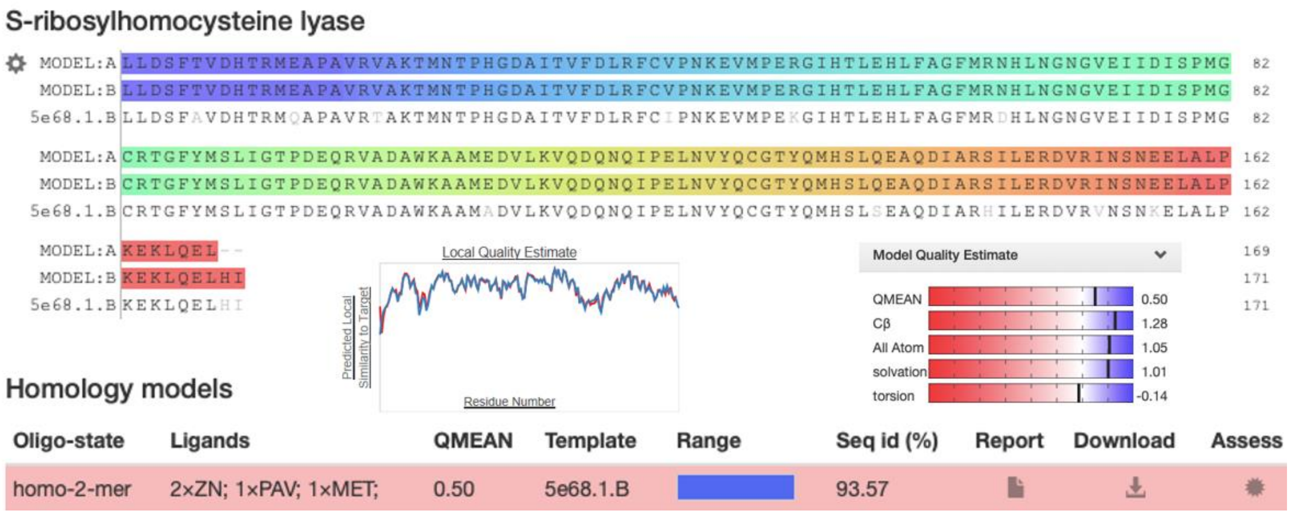

Fig. S1 Information of homology modeling, including alignment of template (5e68) and target sequence, the model quality estimate, for LuxS protein of E. coli.

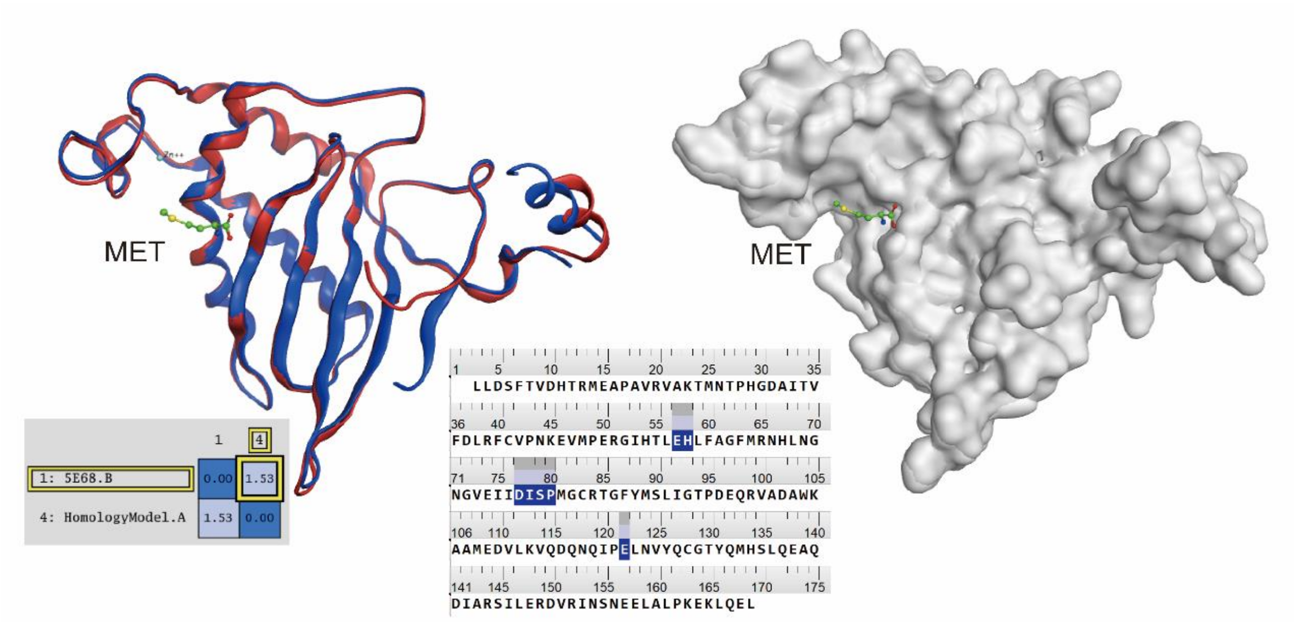

Fig. S2 The 3D model and sequence of LuxS protein of E. coli. The methionine (MET) binding site was marked in blue background.

Table S1 The structure, properties, and diversity of 145 selected compounds.

Structure	SMILE	ID Number	Drug Score	Docking score	MCS Cluster 0.6	MW
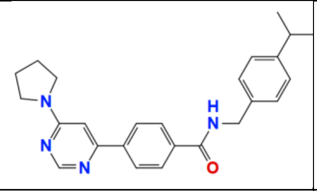	O=C(NCc1ccc(C(C)C)cc1)c1ccc(-c2ncnc(N3CCCC3)c2)cc1	L010-0095	0.01	-11.29	30	400.50
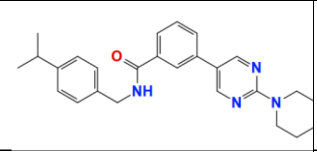	O=C(NCc1ccc(C(C)C)cc1)c1cc(-c2cnc(N3CCCCC3)nc2)ccc1	L368-0245	0.01	-14.20	20	414.50
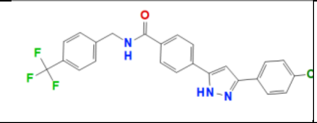	Clc1ccc(-c2n[nH]c(-c3ccc(C(=O)NCc4ccc(C(F)(F)F)cc4)cc3)c2)cc1	V014-2144	0.02	-12.20	51	455.90
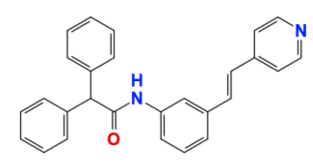	O=C(Nc1cc(/C=C/c2ccncc2)ccc1)C(c1ccccc1)c1ccccc1	8018-3887	0.02	-14.16	0	390.50
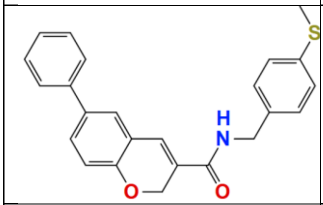	S(C)c1ccc(CNC(=O)C2=Cc3c(OC2)ccc(-c2ccccc2)c3)cc1	L449-1159	0.02	-12.69	33	387.50
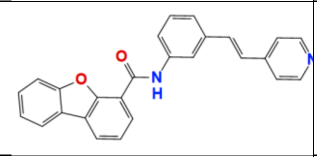	O=C(Nc1cc(/C=C/c2ccncc2)ccc1)c1c2oc3c(c2ccc1)cccc3	8017-8489	0.02	-14.48	0	390.40
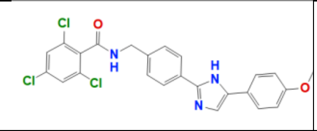	Clc1c(C(=O)NCc2ccc(-c3[nH]c(-c4ccc(OC)cc4)cn3)cc2)c(Cl)cc(Cl)c1	V013-4914	0.02	-12.65	45	486.80
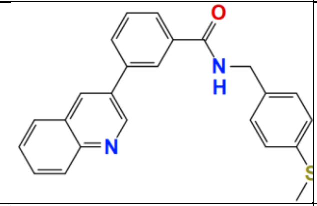	S(C)c1ccc(CNC(=O)c2cc(-c3cnc4c(c3)cccc4)ccc2)cc1	L726-0074	0.02	-14.35	41	384.50
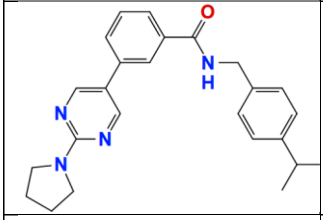	O=C(NCc1ccc(C(C)C)cc1)c1cc(-c2cnc(N3CCCC3)nc2)ccc1	L368-0095	0.02	-13.47	20	400.50
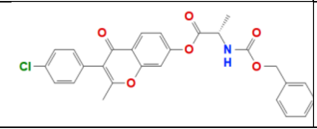	Clc1ccc(C2=C(C)Oc3c(C2=O)ccc(OC(=O)[C@@H](NC(=O)OCc2ccccc2)C)c3)cc1	Y040-8052	0.02	-14.10	17	491.90

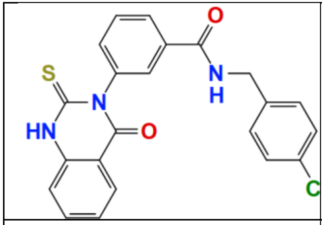	Clc1ccc(CNC(=O)c2cc(N3C(=S)Nc4c(C3=O)cccc4)ccc2)cc1	K284-4314	0.02	-17.58	22	421.90
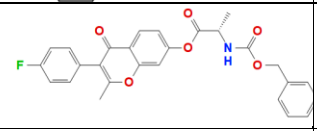	Fc1ccc(C2=C(C)Oc3c(C2=O)ccc(OC(=O)[C@@H](NC(=O)OCc2ccccc2)C)c3)cc1	Y040-8028	0.02	-14.90	17	475.50
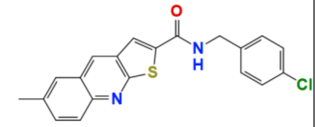	Clc1ccc(CNC(=O)c2sc3nc4c(cc(C)cc4)cc3c2)cc1	G586-0208	0.02	-13.92	26	366.90
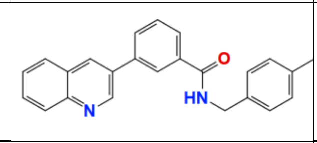	O=C(NCc1ccc(C)cc1)c1cc(-c2cnc3c(c2)cccc3)ccc1	L726-0086	0.03	-13.18	41	352.40
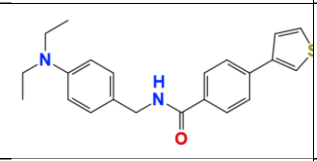	O=C(NCc1ccc(N(CC)CC)cc1)c1ccc(-c2cscc2)cc1	L727-0093	0.03	-11.92	50	364.50
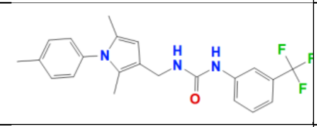	FC(F)(F)c1cc(NC(=O)NCc2c(C)n(c(C)c2)-c2ccc(C)cc2)ccc1	E983-1262	0.04	-16.35	29	401.40
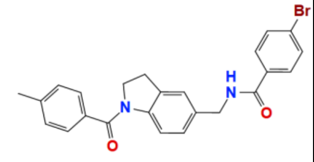	Brc1ccc(C(=O)NCc2cc3c(N(C(=O)c4ccc(C)cc4)CC3)cc2)cc1	G883-0331	0.04	-16.44	4	449.30
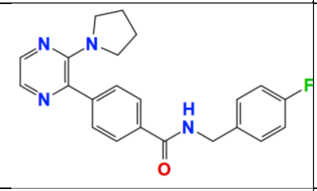	Fc1ccc(CNC(=O)c2ccc(-c3c(N4CCCC4)nccn3)cc2)cc1	P132-0309	0.04	-13.35	28	376.40
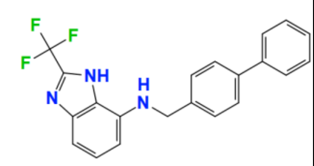	FC(F)(F)c1[nH]c2c(NCc3ccc(-c4ccccc4)cc3)cccc2n1	8017-8462	0.05	-13.57	0	367.40
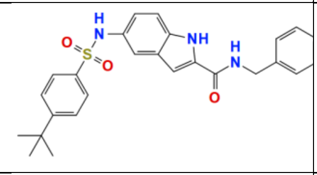	S(=O)(=O)(Nc1cc2c([nH]c(C(=O)NCc3ccccc3)c2)cc1)c1ccc(C(C)(C)C)cc1	V029-0821	0.05	-13.59	13	461.60
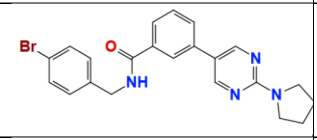	Brc1ccc(CNC(=O)c2cc(-c3cnc(N4CCCC4)nc3)ccc2)cc1	L368-0075	0.05	-14.52	20	437.30
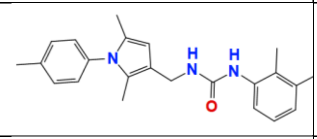	O=C(NCc1c(C)n(c(C)c1)-c1ccc(C)cc1)Nc1c(C)c(C)ccc1	E983-1221	0.05	-13.28	29	361.50
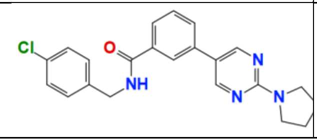	Clc1ccc(CNC(=O)c2cc(-c3cnc(N4CCCC4)nc3)ccc2)cc1	L368-0088	0.05	-14.06	20	392.90

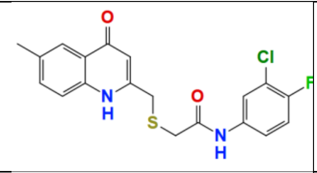	Clc1c(F)ccc(NC(=O)CSCC=2Nc3c(C(=O)C=2)cc(C)cc3)c1	G751-5258	0.05	-13.77	5	390.90
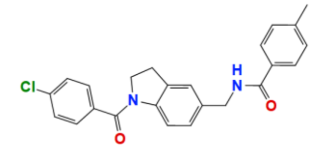	Clc1ccc(C(=O)N2c3c(cc(CNC(=O)c4ccc(C)cc4)cc3)CC2)cc1	G883-0476	0.05	-15.51	4	404.90
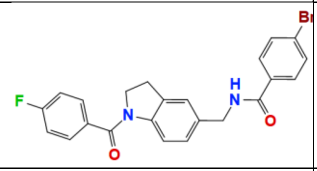	Brc1ccc(C(=O)NCc2cc3c(N(C(=O)c4ccc(F)cc4)CC3)cc2)cc1	G883-0775	0.05	-13.43	4	453.30
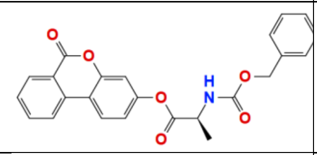	O=C(OCc1ccccc1)N[C@H](C(=O)Oc1cc2OC(=O)c3c(-c2cc1)cccc3)C	Y040-8101	0.06	-14.58	17	417.40
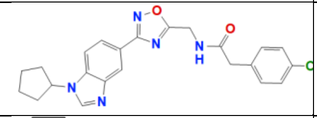	Clc1ccc(CC(=O)NCc2onc(-c3cc4ncn(C5CCCC5)c4cc3)n2)cc1	G945-0615	0.06	-14.02	2	435.90
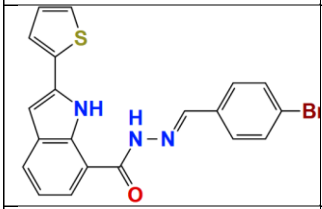	Brc1ccc(/C=N/NC(=O)c2c3[nH]c(-c4sccc4)cc3ccc2)cc1	3286-0440	0.06	-13.10	0	424.30
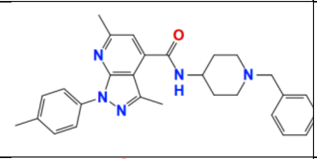	O=C(NC1CCN(Cc2ccccc2)CC1)c1c2c(C)nn(-c3ccc(C)cc3)c2nc(C)c1	E981-1877	0.06	-14.16	0	453.60
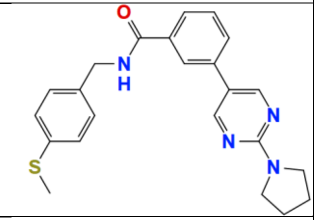	S(C)c1ccc(CNC(=O)c2cc(-c3cnc(N4CCCC4)nc3)ccc2)cc1	L368-0079	0.06	-14.24	20	404.50
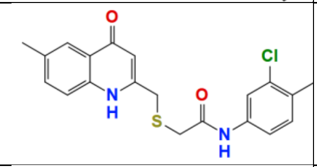	Clc1c(C)ccc(NC(=O)CSCC=2Nc3c(C(=O)C=2)cc(C)cc3)c1	G751-5265	0.06	-14.52	5	386.90
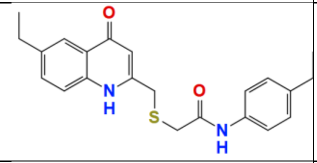	S(CC(=O)Nc1ccc(CC)cc1)CC=1Nc2c(C(=O)C=1)cc(CC)cc2	G751-5495	0.07	-15.00	5	380.50
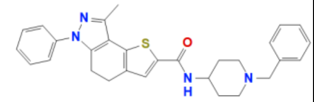	O=C(NC1CCN(Cc2ccccc2)CC1)c1sc2-c3c(C)nn(-c4ccccc4)c3CCc2c1	G519-0167	0.07	-18.87	0	482.60
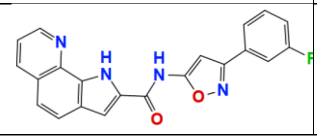	Fc1cc(-c2noc(NC(=O)c3[nH]c4c5ncccc5ccc4c3)c2)ccc1	E950-0302	0.08	-13.24	1	372.40

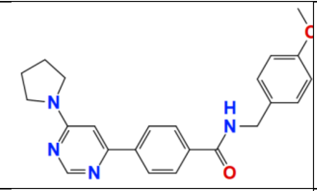	O=C(NCc1ccc(OC)cc1)c1ccc(-c2ncnc(N3CCCC3)c2)cc1	L010-0098	0.08	-12.49	30	388.50
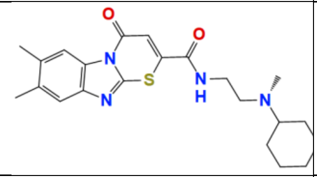	O=C(NCC[N@](C)C1CCCCC1)C=1Sc2n(C(=O)C=1)c1c(n2)cc(C)c(C)c1	F692-0414	0.08	-14.09	0	412.60
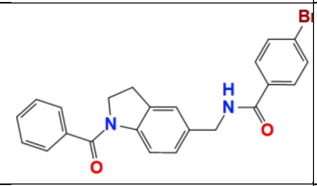	Brc1ccc(C(=O)NCc2cc3c(N(C(=O)c4ccccc4)CC3)cc2)cc1	G883-0627	0.08	-14.86	4	435.30
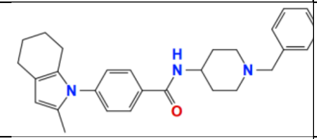	O=C(NC1CCN(Cc2ccccc2)CC1)c1ccc(-n2c(C)cc3c2CCCC3)cc1	E644-0114	0.08	-13.79	53	427.60
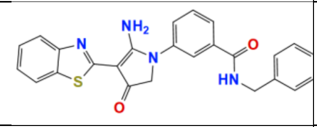	O=C(NCc1ccccc1)c1cc(N2C(N)=C(c3sc4c(n3)cccc4)C(=O)C2)ccc1	F187-0533	0.09	-13.13	3	440.50
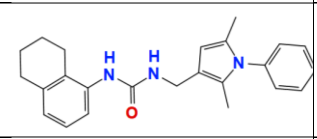	O=C(NCc1c(C)n(c(C)c1)-c1ccccc1)Nc1c2c(ccc1)CCCC2	E983-1808	0.09	-14.49	14	373.50
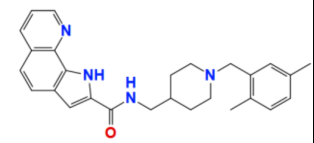	O=C(NCC1CCN(Cc2c(C)ccc(C)c2)CC1)c1[nH]c2c3ncccc3ccc2c1	E950-0382	0.09	-13.95	1	426.60
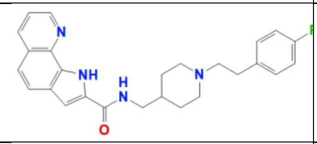	Fc1ccc(CCN2CCC(CNC(=O)c3[nH]c4c5ncccc5ccc4c3)CC2)cc1	E950-0417	0.09	-12.39	1	430.50
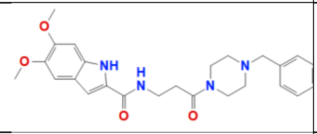	O=C(CCNC(=O)c1[nH]c2c(cc(OC)c(OC)c2)c1)N1CCN(Cc2ccccc2)CC1	Y040-8930	0.09	-15.29	8	450.50
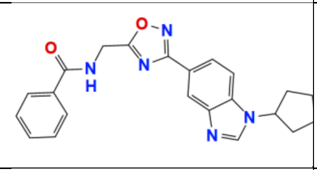	O=C(NCc1onc(-c2cc3ncn(C4CCCC4)c3cc2)n1)c1ccccc1	G945-0601	0.10	-11.82	2	387.40
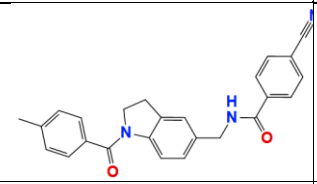	O=C(NCc1cc2c(N(C(=O)c3ccc(C)cc3)CC2)cc1)c1ccc(C#N)cc1	G883-0304	0.10	-14.23	4	395.50
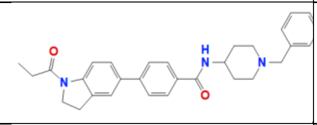	O=C(CC)N1c2c(cc(-c3ccc(C(=O)NC4CCN(Cc5ccccc5)CC4)cc3)cc2)CC1	L413-0101	0.10	-13.89	16	467.60
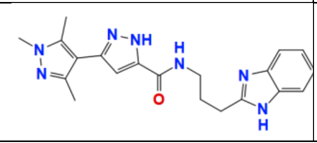	O=C(NCCCc1[nH]c2c(n1)cccc2)c1[nH]nc(-c2c(C)n(C)nc2C)c1	Y042-6613	0.10	-15.54	43	377.40

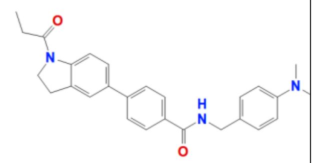	O=C(CC)N1c2c(cc(-c3ccc(C(=O)NCc4ccc(N(C)C)cc4)cc3)cc2)CC1	L413-0180	0.10	-13.08	21	427.50
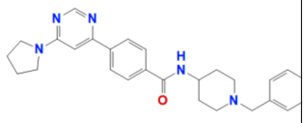	O=C(NC1CCN(Cc2ccccc2)CC1)c1ccc(-c2ncnc(N3CCCC3)c2)cc1	L010-0050	0.11	-16.75	30	441.60
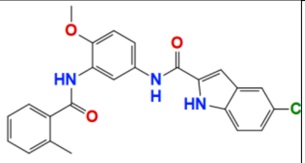	Clc1cc2c([nH]c(C(=O)Nc3cc(NC(=O)c4c(C)cccc4)c(OC)cc3)c2)cc1	F603-0876	0.11	-13.97	9	433.90
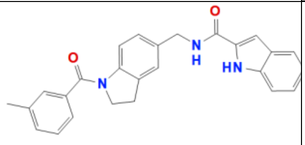	O=C(NCc1cc2c(N(C(=O)c3cc(C)ccc3)CC2)cc1)c1[nH]c2c(c1)cccc2	G883-1589	0.11	-14.17	4	409.50
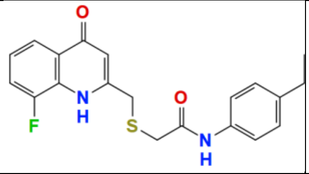	S(CC(=O)Nc1ccc(CC)cc1)CC=1Nc2c(F)cccc2C(=O)C=1	G751-5415	0.11	-15.61	5	370.40
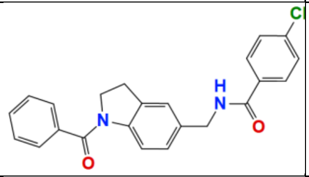	Clc1ccc(C(=O)NCc2cc3c(N(C(=O)c4ccccc4)CC3)cc2)cc1	G883-0595	0.11	-15.43	4	390.90
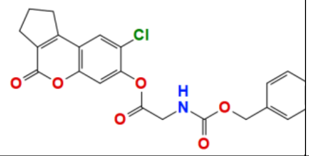	Clc1c(OC(=O)CNC(=O)OCc2ccccc2)cc2OC(=O)C3=C(c2c1)CCC3	Y041-2475	0.12	-13.79	17	427.80
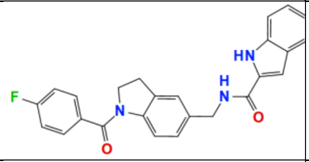	Fc1ccc(C(=O)N2c3c(cc(CNC(=O)c4[nH]c5c(c4)cccc5)cc3)CC2)cc1	G883-0849	0.12	-14.26	4	413.40
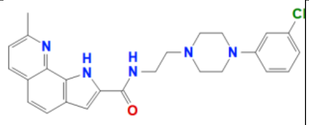	Clc1cc(N2CCN(CCNC(=O)c3[nH]c4c5nc(C)ccc5ccc4c3)CC2)ccc1	E941-0195	0.12	-14.11	1	448.00
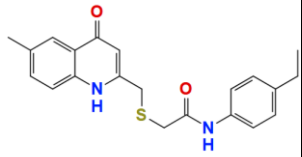	S(CC(=O)Nc1ccc(CC)cc1)CC=1Nc2c(C(=O)C=1)cc(C)cc2	G751-5255	0.12	-15.07	5	366.50
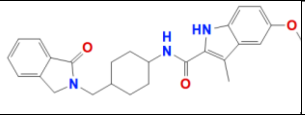	O=C(NC1CCC(CN2C(=O)c3c(cccc3)C2)CC1)c1c(C)c2c([nH]1)ccc(OC)c2	D753-0186	0.12	-14.05	0	431.50
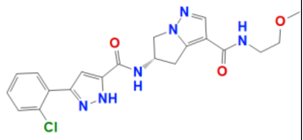	Clc1c(-c2n[nH]c(C(=O)N[C@@H]3Cn4ncc(C(=O)NCCOC)c4C3)c2)cccc1	SB68-0718	0.12	-14.29	0	428.90
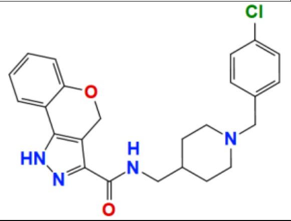	Clc1ccc(CN2CCC(CNC(=O)c3n[nH]c4-c5c(OCc34)cccc5)CC2)cc1	G365-0523	0.13	-14.81	0	436.90

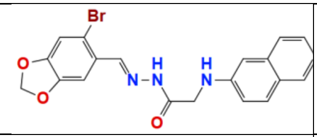	Brc1c(/C=N/NC(=O)CNc2cc3c(cc2)cccc3)cc2OCOc2c1	8004-4282	0.13	-13.13	36	426.30
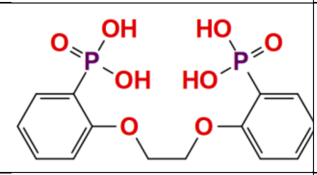	P(=O)(O)(O)c1c(OCCOc2c(P(=O)(O)O)cccc2)cccc1	4470-0385	0.13	-14.70	0	374.20
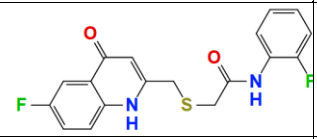	S(CC(=O)Nc1c(F)cccc1)CC=1Nc2c(C(=O)C=1)cc(F)cc2	G751-2322	0.14	-13.97	5	360.40
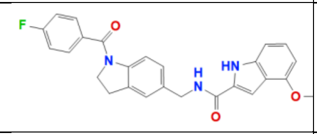	Fc1ccc(C(=O)N2c3c(cc(CNC(=O)c4[nH]c5c(c(OC)ccc5)c4)cc3)CC2)cc1	G883-0887	0.14	-14.17	4	443.50
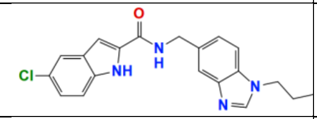	Clc1cc2c([nH]c(C(=O)NCc3cc4ncn(CCC)c4cc3)c2)cc1	M414-3326	0.14	-15.32	13	366.80
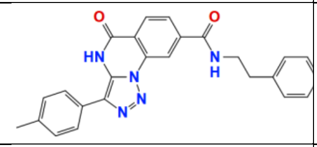	O=C(NCCc1ccccc1)c1cc2-n3nnc(-c4ccc(C)cc4)c3NC(=O)c2cc1	E543-0259	0.14	-14.51	35	423.50
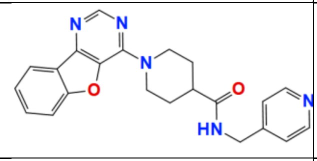	O=C(NCc1ccncc1)C1CCN(c2ncnc3c2oc2c3cccc2)CC1	L928-0081	0.16	-14.93	0	387.40
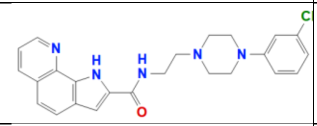	Clc1cc(N2CCN(CCNC(=O)c3[nH]c4c5ncccc5ccc4c3)CC2)ccc1	E950-0195	0.16	-12.70	1	433.90
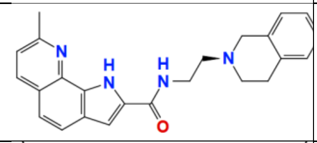	O=C(NCC[N@]1Cc2c(cccc2)CC1)c1[nH]c2c3nc(C)ccc3ccc2c1	E941-0193	0.16	-13.18	1	384.50
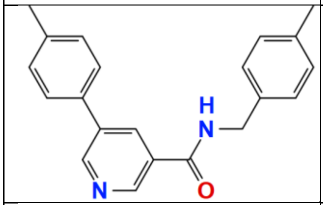	O=C(NCc1ccc(C)cc1)c1cc(-c2ccc(C)cc2)cnc1	L428-0392	0.17	-13.04	24	316.40
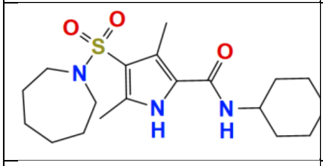	S(=O)(=O)(N1CCCCCC1)c1c(C)c(C(=O)NC2CCCCC2)[nH]c1C	F379-1612	0.18	-15.18	0	381.50
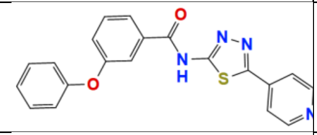	O=C(Nc1sc(-c2ccncc2)nn1)c1cc(Oc2ccccc2)ccc1	Y200-0134	0.18	-12.92	0	374.40
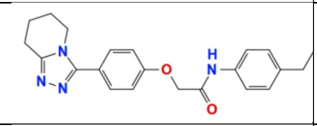	O=C(Nc1ccc(CC)cc1)COc1ccc(-c2n3c(nn2)CCCC3)cc1	S346-0296	0.18	-15.66	0	376.50
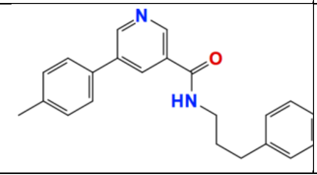	O=C(NCCCc1ccccc1)c1cc(-c2ccc(C)cc2)cnc1	L428-0390	0.18	-12.69	46	330.40

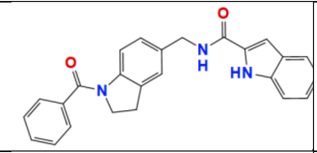	O=C(NCc1cc2c(N(C(=O)c3ccccc3)CC2)cc1)c1[nH]c2c(c1)cccc2	G883-0701	0.18	-14.12	4	395.50
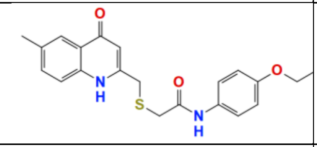	S(CC(=O)Nc1ccc(OCC)cc1)CC=1Nc2c(C(=O)C=1)cc(C)cc2	G751-5250	0.18	-13.27	5	382.50
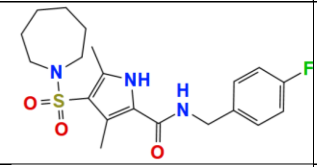	S(=O)(=O)(N1CCCCCC1)c1c(C)c(C(=O)NCc2ccc(F)cc2)[nH]c1C	F379-1712	0.19	-10.59	7	407.50
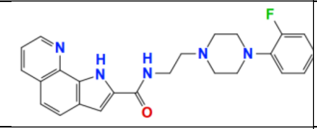	Fc1c(N2CCN(CCNC(=O)c3[nH]c4c5ncccc5ccc4c3)CC2)cccc1	E950-0198	0.19	-12.94	1	417.50
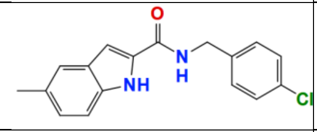	Clc1ccc(CNC(=O)c2[nH]c3c(c2)cc(C)cc3)cc1	D291-0905	0.19	-12.64	13	298.80
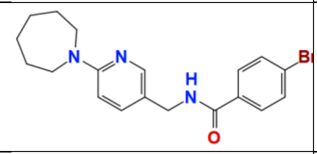	Brc1ccc(C(=O)NCc2cnc(N3CCCCCC3)cc2)cc1	M626-0702	0.19	-12.84	48	388.30
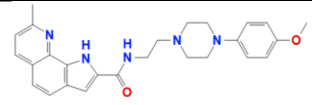	O=C(NCCN1CCN(c2ccc(OC)cc2)CC1)c1[nH]c2c3nc(C)ccc3ccc2c1	E941-0156	0.19	-13.78	1	443.50
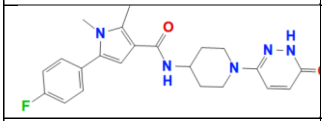	Fc1ccc(-c2n(C)c(C)c(C(=O)NC3CCN(C4=NNC(=O)C=C4)CC3)c2)cc1	CM3124-0506	0.20	-12.97	23	409.50
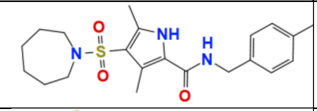	S(=O)(=O)(N1CCCCCC1)c1c(C)c(C(=O)NCc2ccc(C)cc2)[nH]c1C	F379-1641	0.20	-12.80	7	403.50
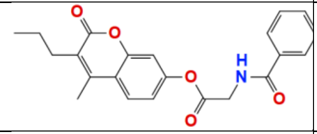	O=C(Oc1cc2OC(=O)C(CCC)=C(C)c2cc1)CNC(=O)c1ccccc1	Y040-2389	0.23	-15.31	37	379.40
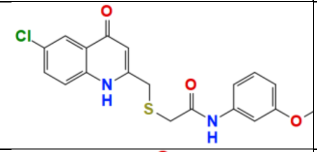	Clc1cc2C(=O)C=C(CSCC(=O)Nc3cc(OC)ccc3)Nc2cc1	G751-1990	0.23	-14.44	5	388.90
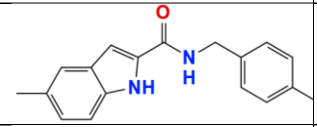	O=C(NCc1ccc(C)cc1)c1[nH]c2c(c1)cc(C)cc2	D291-0890	0.23	-14.09	13	278.30
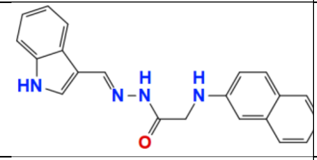	O=C(N/N=C/c1c2c([nH]c1)cccc2)CNc1cc2c(cc1)cccc2	2181-0039	0.23	-10.48	32	342.40
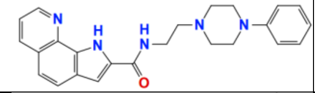	O=C(NCCN1CCN(c2ccccc2)CC1)c1[nH]c2c3ncccc3ccc2c1	E950-0345	0.24	-16.11	1	399.50
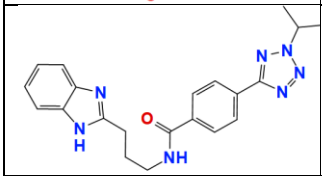	O=C(NCCCc1[nH]c2c(n1)cccc2)c1ccc(-c2nn(C(C)C)nn2)cc1	Y043-4731	0.24	-15.68	43	389.50

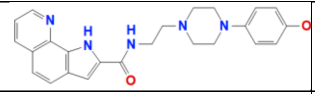	O=C(NCCN1CCN(c2ccc(OC)cc2)CC1)c1[nH]c2c3ncccc3ccc2c1	E950-0156	0.24	-14.05	1	429.50
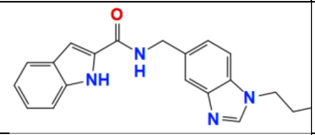	O=C(NCc1cc2ncn(CCC)c2cc1)c1[nH]c2c(c1)cccc2	M414-3315	0.25	-13.89	13	332.40
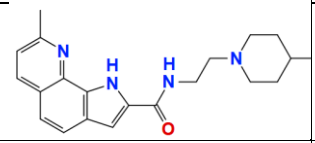	O=C(NCCN1CCC(C)CC1)c1[nH]c2c3nc(C)ccc3ccc2c1	E941-0343	0.25	-12.94	1	350.50
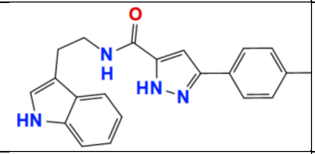	O=C(NCCc1c2c([nH]c1)cccc2)c1[nH]nc(-c2ccc(C)cc2)c1	Y040-3265	0.26	-13.43	0	344.40
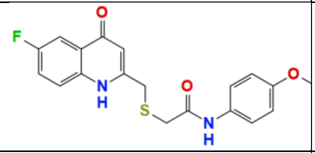	S(CC(=O)Nc1ccc(OC)cc1)CC=1Nc2c(C(=O)C=1)cc(F)cc2	G751-2329	0.27	-14.93	5	372.40
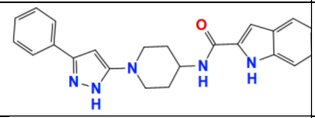	O=C(NC1CCN(c2[nH]nc(-c3ccccc3)c2)CC1)c1[nH]c2c(c1)cccc2	P805-0587	0.27	-14.77	0	385.50
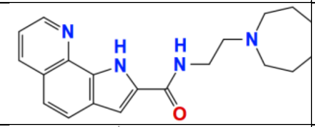	O=C(NCCN1CCCCCC1)c1[nH]c2c3ncccc3ccc2c1	E950-0265	0.29	-12.85	1	336.40
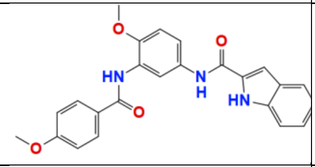	O=C(Nc1cc(NC(=O)c2ccc(OC)cc2)c(OC)cc1)c1[nH]c2c(c1)cccc2	F603-0744	0.30	-16.94	9	415.40
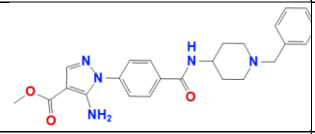	O=C(OC)c1c(N)n(-c2ccc(C(=O)NC3CCN(Cc4ccccc4)CC3)cc2)nc1	G892-1103	0.30	-15.75	53	433.50
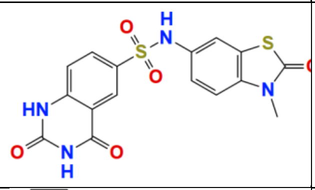	S(=O)(=O)(Nc1cc2SC(=O)N(C)c2cc1)c1cc2C(=O)NC(=O)Nc2cc1	C301-8875	0.31	-17.19	0	404.40
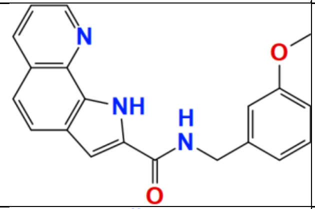	O=C(NCc1cc(OC)ccc1)c1[nH]c2c3ncccc3ccc2c1	E950-0210	0.32	-13.05	1	331.40
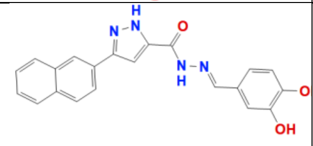	O=C(N/N=C/c1cc(O)c(O)cc1)c1[nH]nc(-c2cc3c(cc2)cccc3)c1	3254-3286	0.32	-14.66	12	372.40
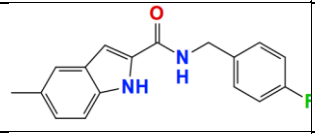	Fc1ccc(CNC(=O)c2[nH]c3c(c2)cc(C)cc3)cc1	D291-0867	0.32	-12.66	13	282.30
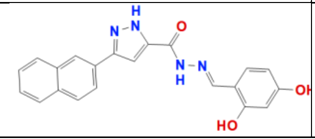	O=C(N/N=C/c1c(O)cc(O)cc1)c1[nH]nc(-c2cc3c(cc2)cccc3)c1	2323-2332	0.33	-14.40	12	372.40

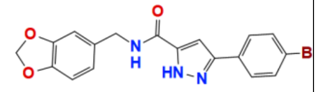	Brc1ccc(-c2n[nH]c(C(=O)NCc3cc4OCOc4cc3)c2)cc1	E822-0067	0.33	-14.81	27	400.20
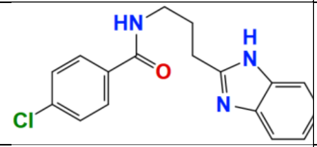	Clc1ccc(C(=O)NCCCc2[nH]c3c(n2)cccc3)cc1	C174-0934	0.34	-13.33	43	313.80
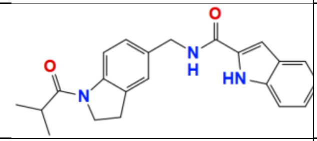	O=C(C(C)C)N1c2c(cc(CNC(=O)c3[nH]c4c(c3)cccc4)cc2)CC1	G883-2010	0.35	-11.06	13	361.40
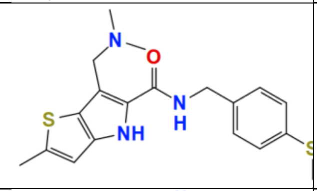	S(C)c1ccc(CNC(=O)c2c(CN(C)C)c3sc(C)cc3[nH]2)cc1	M372-1241	0.35	-13.20	15	373.50
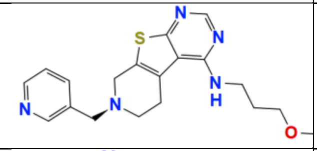	O(CCCNc1ncnc2sc3c(c12)CC[N@@](Cc1cnccc1)C3)C	S324-0240	0.35	-17.48	11	369.50
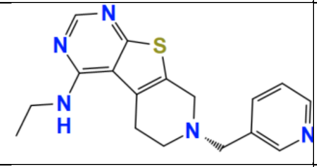	N(CC)c1ncnc2sc3c(c12)CC[N@@](Cc1cnccc1)C3	S324-0175	0.35	-18.32	11	325.40
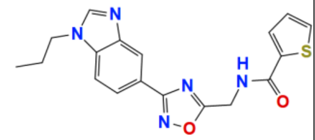	O=C(NCc1onc(-c2cc3ncn(CCC)c3cc2)n1)c1sccc1	G945-0335	0.36	-15.55	0	367.40
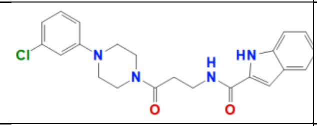	Clc1cc(N2CCN(C(=O)CCNC(=O)c3[nH]c4c(c3)cccc4)CC2)ccc1	Y040-8976	0.36	-13.32	8	410.90
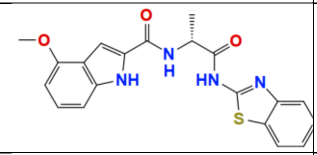	O=C(Nc1sc2c(n1)cccc2)[C@H](NC(=O)c1[nH]c2c(c(OC)ccc2)c1)C	Y040-6167	0.37	-12.42	42	394.40
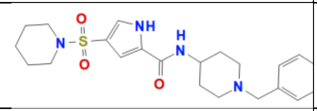	S(=O)(=O)(N1CCCCC1)c1cc(C(=O)NC2CCN(Cc3ccccc3)CC2)[nH]c1	E511-4137	0.38	-12.86	38	430.60
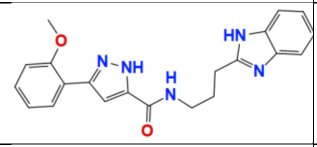	O=C(NCCCc1[nH]c2c(n1)cccc2)c1[nH]nc(-c2c(OC)cccc2)c1	Y041-7661	0.40	-17.52	43	375.40
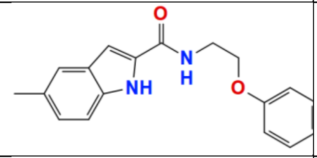	O=C(NCCOc1ccccc1)c1[nH]c2c(c1)cc(C)cc2	D291-0859	0.40	-10.31	47	294.30
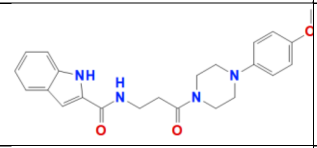	O=C(CCNC(=O)c1[nH]c2c(c1)cccc2)N1CCN(c2ccc(OC)cc2)CC1	Y040-8988	0.40	-14.03	8	406.50
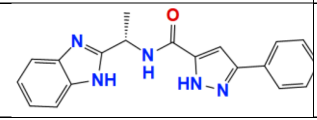	O=C(N[C@@H](C)c1[nH]c2c(n1)cccc2)c1[nH]nc(-c2ccccc2)c1	Y042-6589	0.41	-15.81	25	331.40

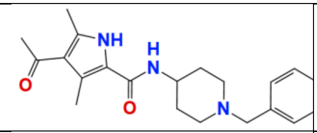	O=C(NC1CCN(Cc2ccccc2)CC1)c1c(C)c(C(=O)C)c(C)[nH]1	C444-0430	0.43	-10.66	49	353.50
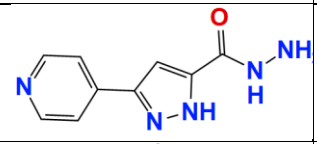	O=C(NN)c1[nH]nc(-c2ccncc2)c1	S553-0291	0.43	-17.63	0	203.20
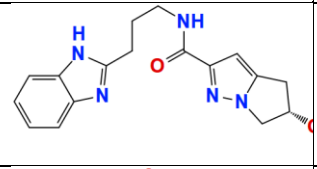	O=C(NCCCc1[nH]c2c(n1)cccc2)c1nn2c(c1)C[C@H](OC)C2	SB69-0037	0.44	-14.34	43	339.40
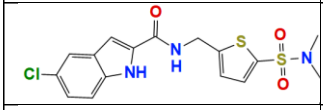	Clc1cc2c([nH]c(C(=O)NCc3sc(S(=O)(=O)N(C)C)cc3)c2)cc1	L470-1245	0.44	-15.19	18	397.90
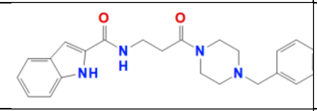	O=C(CCNC(=O)c1[nH]c2c(c1)cccc2)N1CCN(Cc2ccccc2)CC1	Y040-8987	0.44	-13.57	8	390.50
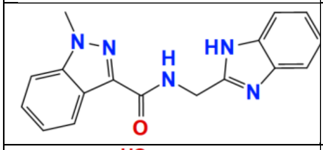	O=C(NCc1[nH]c2c(n1)cccc2)c1nn(C)c2c1cccc2	Y041-7625	0.45	-13.95	25	305.30
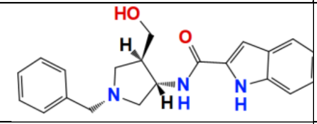	O=C(N[C@H]1[C@H](CO)C[N@@](Cc2ccccc2)C1)c1[nH]c2c(c1)cccc2	S823-0933	0.45	-11.47	0	349.40
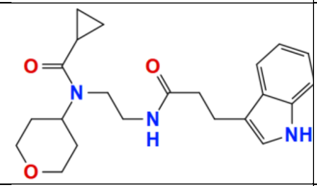	O=C(NCCN(C(=O)C1CC1)C1CCOCC1)CCc1c2c([nH]c1)cccc2	SA59-1555	0.46	-14.92	0	383.50
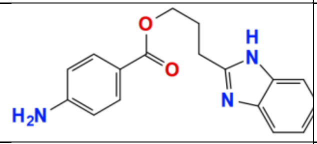	O=C(OCCCc1[nH]c2c(n1)cccc2)c1ccc(N)cc1	0812-1018	0.46	-13.27	0	295.30
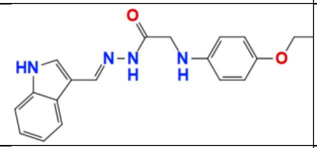	O=C(N/N=C/c1c2c([nH]c1)cccc2)CNc1ccc(OCC)cc1	1761-1352	0.46	-16.32	32	336.40
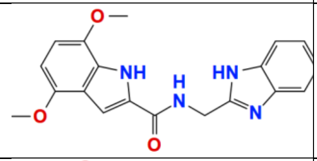	O=C(NCc1[nH]c2c(n1)cccc2)c1[nH]c2c(OC)ccc(OC)c2c1	Y043-4179	0.47	-11.09	0	350.40
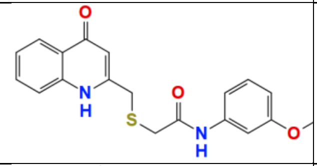	S(CC(=O)Nc1cc(OC)ccc1)CC=1Nc2c(C(=O)C=1)cccc2	G751-0153	0.48	-14.10	5	354.40
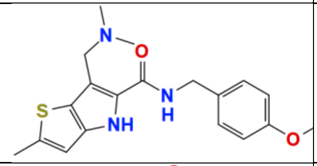	O=C(NCc1ccc(OC)cc1)c1c(CN(C)C)c2sc(C)cc2[nH]1	M372-1113	0.48	-13.18	15	357.50
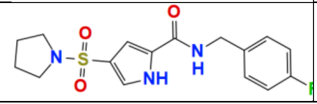	S(=O)(=O)(N1CCCC1)c1cc(C(=O)NCc2ccc(F)cc2)[nH]c1	E511-4033	0.51	-13.98	7	351.40

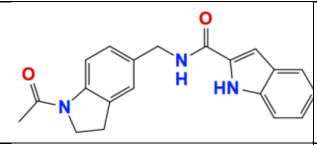	O=C(C)N1c2c(cc(CNC(=O)c3[nH]c4c(c3)cccc4)cc2)CC1	G883-0109	0.52	-11.01	13	333.40
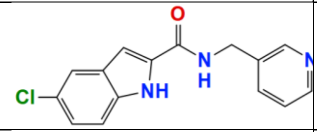	Clc1cc2c([nH]c(C(=O)NCc3cnccc3)c2)cc1	Z601-7646	0.52	-13.47	39	285.70
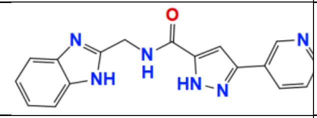	O=C(NCc1[nH]c2c(n1)cccc2)c1[nH]nc(-c2cnccc2)c1	Y042-6989	0.57	-11.89	25	318.30
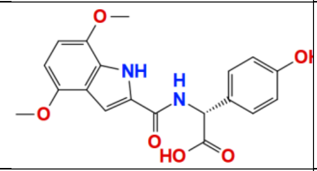	O=C(O)[C@H](NC(=O)c1[nH]c2c(OC)ccc(OC)c2c1)c1ccc(O)cc1	D715-0786	0.58	-10.51	13	370.40
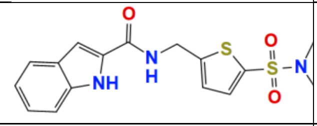	S(=O)(=O)(N(C)C)c1sc(CNC(=O)c2[nH]c3c(c2)cccc3)cc1	L470-1233	0.58	-14.71	18	363.50
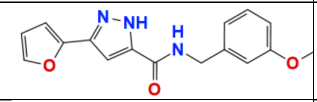	O=C(NCc1cc(OC)ccc1)c1[nH]nc(-c2occc2)c1	E822-1215	0.59	-12.92	0	297.30
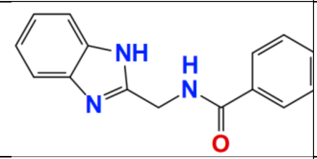	O=C(NCc1[nH]c2c(n1)cccc2)c1ccccc1	0180-0358	0.63	-12.16	25	251.30
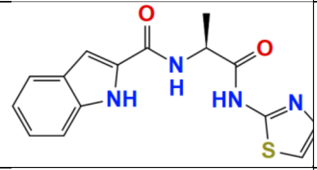	O=C(Nc1sccn1)[C@@H](NC(=O)c1[nH]c2c(c1)cccc2)C	Y040-3218	0.64	-10.50	42	314.40
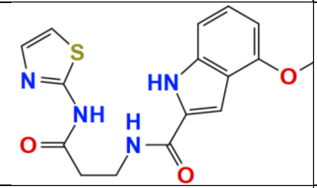	O=C(Nc1sccn1)CCNC(=O)c1[nH]c2c(c(OC)ccc2)c1	Y040-9862	0.65	-12.15	8	344.40
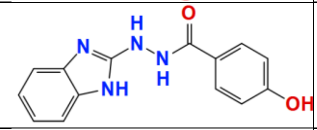	O=C(NNc1[nH]c2c(n1)cccc2)c1ccc(O)cc1	8017-9549	0.66	-11.51	0	268.30
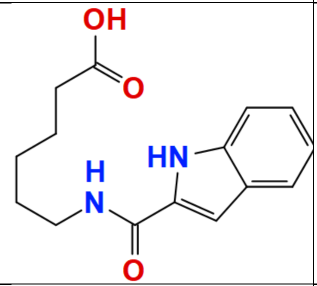	O=C(O)CCCCCNC(=O)c1[nH]c2c(c1)cccc2	Y040-9185	0.67	-18.10	40	274.30
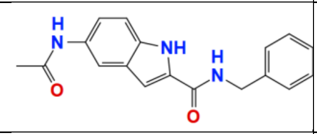	O=C(Nc1cc2c([nH]c(C(=O)NCc3ccccc3)c2)cc1)C	V029-0772	0.68	-13.26	13	307.30
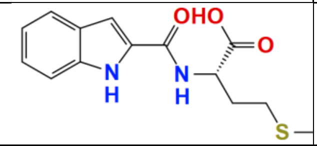	S(CC[C@H](NC(=O)c1[nH]c2c(c1)cccc2)C(=O)O)C	Y040-3091	0.76	-10.70	47	292.40

Table S2. The primer sequences used in qRT-PCR

Genes	sequence (5’ to 3’)	Product size (bp)
gapA-F	CCAGGACATCGTTTCCAAC	103
gapA-R	GGTGGTCATCAGACCTTCG	103
luxS-F	GAAAACAATGAACACCCCGCATGG	92
luxS-R	TCCCTCTTTCTGGCATCACTTCTTTG	92
